# **Understanding the Early Evolutionary Stages of a Tandem** *Drosophila****melanogaster*-Specific Gene Family: A Structural and Functional Population Study**

**DOI:** 10.1093/molbev/msaa109

**Published:** 2020-05-02

**Authors:** Bryan D Clifton, Jamie Jimenez, Ashlyn Kimura, Zeinab Chahine, Pablo Librado, Alejandro Sánchez-Gracia, Mashya Abbassi, Francisco Carranza, Carolus Chan, Marcella Marchetti, Wanting Zhang, Mijuan Shi, Christine Vu, Shudan Yeh, Laura Fanti, Xiao-Qin Xia, Julio Rozas, José M Ranz

**Affiliations:** m1 Department of Ecology and Evolutionary Biology, University of California Irvine, Irvine, CA; m2 Laboratoire AMIS CNRS UMR 5288, Faculté de Médicine de Purpan, Université Paul Sabatier, Toulouse, France; m3 Departament de Genètica, Microbiologia i Estadistica, Universitat de Barcelona, Barcelona, Spain; m4 Institut de Recerca de la Biodiversitat, Universitat de Barcelona, Barcelona, Spain; m5 Istituto Pasteur Italia, Fondazione Cenci-Bolognetti, Rome, Italy; m6 Department of Biology and Biotechnology “C. Darwin”, Sapienza University of Rome, Rome, Italy; m7 Institute of Hydrobiology, Chinese Academy of Sciences, Wuhan, Hubei Province, China; m8 Department of Life Sciences, National Central University, Taoyuan City, Zhongli District, Taiwan

**Keywords:** complex genomic regions, tandem multigene families, CNV, expression variation, gene conversion, sexual selection

## Abstract

Gene families underlie genetic innovation and phenotypic diversification. However, our understanding of the early genomic and functional evolution of tandemly arranged gene families remains incomplete as paralog sequence similarity hinders their accurate characterization. The *Drosophila melanogaster*-specific gene family *Sdic* is tandemly repeated and impacts sperm competition. We scrutinized *Sdic* in 20 geographically diverse populations using reference-quality genome assemblies, read-depth methodologies, and qPCR, finding that ∼90% of the individuals harbor 3–7 copies as well as evidence of population differentiation. In strains with reliable gene annotations, copy number variation (CNV) and differential transposable element insertions distinguish one structurally distinct version of the *Sdic* region per strain. All 31 annotated copies featured protein-coding potential and, based on the protein variant encoded, were categorized into 13 paratypes differing in their 3′ ends, with 3–5 paratypes coexisting in any strain examined. Despite widespread gene conversion, the only copy present in all strains has functionally diverged at both coding and regulatory levels under positive selection. Contrary to artificial tandem duplications of the *Sdic* region that resulted in increased male expression, CNV in cosmopolitan strains did not correlate with expression levels, likely as a result of differential genome modifier composition. Duplicating the region did not enhance sperm competitiveness, suggesting a fitness cost at high expression levels or a plateau effect. Beyond facilitating a minimally optimal expression level, *Sdic* CNV acts as a catalyst of protein and regulatory diversity, showcasing a possible evolutionary path recently formed tandem multigene families can follow toward long-term consolidation in eukaryotic genomes.

## Introduction

Structural variants have been largely overlooked in genetic variation surveys, limiting our understanding on the genetic basis of phenotypic change (Feyereisen et al. 2015; Huddleston and Eichler 2016; Chakraborty et al. 2019). Structural variants include >50-nt-long duplications and deletions, transpositions, inversions, and translocations. Complex genomic regions, those that exhibit unusually high levels of structural variation often in the form multiple copies of particular, high identity sequences generated by some kind of duplicative mechanism, are predominantly affected by this oversight. Accordingly, these regions are often grossly misassembled or absent altogether in reference genome assemblies (Hollox 2012; Ranz and Clifton 2019). This in turn precludes their accurate genomic and functional characterization, which is relevant given the close interplay between these regions, evolutionary change, and disease (Dennis and Eichler 2016). This interplay arises from the proclivity of complex genomic regions to structural remodeling (Hurles 2004; Hollox 2012), often resulting in marked copy number variation (CNV) patterns for the encompassed genes (Sudmant et al. 2010; Jiang et al. 2012; Carpenter et al. 2015) and in the formation of new gene entities with chimeric or defective features (Dennis et al. 2012; Nuttle et al. 2016; Fiddes et al. 2018). Despite the potential of these genomic regions to impact the phenotype and organismal fitness (Hollox 2008; Jugulam et al. 2014; Chakraborty et al. 2019), our understanding of how they evolve remains largely incomplete.

To date, most complex genomic regions characterized molecularly have been linked to traits associated with viability and fecundity (Dennis et al. 2017; Chakraborty et al. 2019) as opposed with reproductive success, that is, to traits targeted by sexual selection rather than by natural selection (Darwin 1871). A form of sexual selection, sperm competition, biases fertilization at the postcopulatory level in numerous species groups (Parker 1970; Birkhead 1998). Among the few genetic factors known to affect sperm competition (Civetta and Ranz 2019), there is one that resides within a complex region of the *Drosophila melanogaster* euchromatin: the tandem multigene family *Sdic. Sdic* is absent in the rest of the genus *Drosophila*, having originated at some point in the *D. melanogaster* lineage after diverging from the *simulans* clade ∼1.4 Ma (Nurminsky et al. 1998; Obbard et al. 2012).

The original *Sdic* gene resulted from a segmental duplication on the *X* chromosome spanning two adjacent genes, *sw* and *AnxB10*, which fused through a set of deletions while accommodating multiple nucleotide substitutions. Subsequently, this chimeric entity underwent a tandem expansion (Nurminsky et al. 1998). The repetitive nature of *Sdic* and the high sequence similarity among the resident paralogs make this region prone to recurrent nonallelic homologous recombination (NAHR) events, that is, unequal crossing over, which should result in contractions and expansions of the tandem array (Hastings et al. 2009). Thus, the organization of the *Sdic* region in the *D. melanogaster* reference strain, which includes six copies of a repeat unit, spanning in total ∼46 kb (Clifton et al. 2017), might just be a nonrepresentative state within the actual breadth in copy number (CN) in natural populations. In fact, the CN distribution at the *Sdic* region is unknown, as are the occurrence of other structural changes (e.g., transposable element—TE—insertions) and the frequency of structurally distinct versions of the region. Also unknown is the extent to which *Sdic* CNV can impact expression levels, as often assumed after tandem duplication events (Kondrashov et al. 2002; Kondrashov 2010), or can act as a catalyst for protein diversification (Traherne et al. 2010), or both. In fact, without this crucial information, it is not feasible to determine whether putative expression changes mirroring alterations in *Sdic* CN actually impact sperm competitive ability. Further, it is unclear whether the patterns of gene conversion and overall sequence conservation documented across the *Sdic* copies in the reference strain hold in strains representing other populations of *D. melanogaster*. Overall, *Sdic*, offers the opportunity to investigate different levels of change and their consequences at the early stages of a recently expanded multigene family, which has been typically neglected despite its importance to understand the fate of gene duplicates and the origin of new gene functions (Kondrashov 2010; Katju and Bergthorsson 2013; Long et al. 2013; Cardoso-Moreira et al. 2016; Naseeb et al. 2017; Rogers et al. 2017).

We have analyzed the *Sdic* region at the genetic, functional, and phenotypic levels using two panels of strains with diverse geographical origin, including the ancestral sub-Saharan distribution range of *D. melanogaster* (Begun and Aquadro 1993), and other synthetic strains harboring complete duplications of the *Sdic* region. We aim at: 1) gauging the breadth of *Sdic* CNV in different parts of the world using the annotation of the region in reference-quality genome assemblies, qPCR assays, and read-depth algorithms suitable for analyzing Illumina sequencing reads; 2) evaluating the role of positive selection in explaining the sequence evolution at the coding and noncoding levels of this tandemly arranged multigene family, as well as the relevance of gene conversion; 3) determining by qRT–PCR assays the extent to which CNV translates into expression variation in natural populations and genome-edited strains that allow control of genomic background differences; and 4) testing whether increased *Sdic* expression correlates with varying sperm competitive ability using different genetic modifications of the *Sdic* region. While answering some of these questions, we also found that a fraction of reference-quality assemblies generated with single-molecule real-time (SMRT) and Nanopore sequencing technologies still do not faithfully recapitulate the organization of the *Sdic* region.

## Results

### Naturally Occurring CNV in the *Sdic* Region

To generate a global portrait of *Sdic* CNV in *D. melanogaster*, we examined two different panels of strains. First, we focused on a panel of 15 strains (eight from the Americas; two from Africa; and five from Eurasia and the Middle East; supplementary tables S1 and S2, Supplementary Material online) for which female-derived reference-quality assemblies have been generated (Chakraborty et al. 2018, 2019). These assemblies offer the opportunity to parse patterns of additional structural variation, including inversions and TE insertions, in addition to calibrate two other approaches to estimate CNV: qPCR and read-depth analysis. Second, using read-depth analysis, we extended our characterization of *Sdic* CNV to a panel that includes strains from populations derived from five different locations around the globe in order to estimate population parameters that can help uncover *Sdic*’s evolutionary mode of structural remodeling across *D. melanogaster*’s entire range.

#### Individual D. melanogaster Populations Consist of Various Numbers of Sdic Copies

We annotated the *Sdic* region in 14 de novo, reference-quality genome assemblies scaffolded with SMRT sequencing reads (Chakraborty et al. 2018, 2019). Thirteen of them correspond to strains from the Drosophila Synthetic Population Resource (DSPR) and are virtually isogenic (King, Merkes, et al. 2012); the 14th strain is the commonly used laboratory, wild-type stock OR-R. The structural and sequence features of the region were compared across assemblies against its updated reconstruction in the ISO-1 reference strain, which is based on the sequence of the GCA_000778455 assembly (Berlin et al. 2015) as opposed to that of the Release 6 (dos Santos et al. 2015), as the former more accurately recapitulates the *Sdic* region (Clifton et al. 2017). This prevents inaccurate inferences about the type and magnitude of genetic differences across the strains considered (Supplementary Text).

**Fig. 1.**
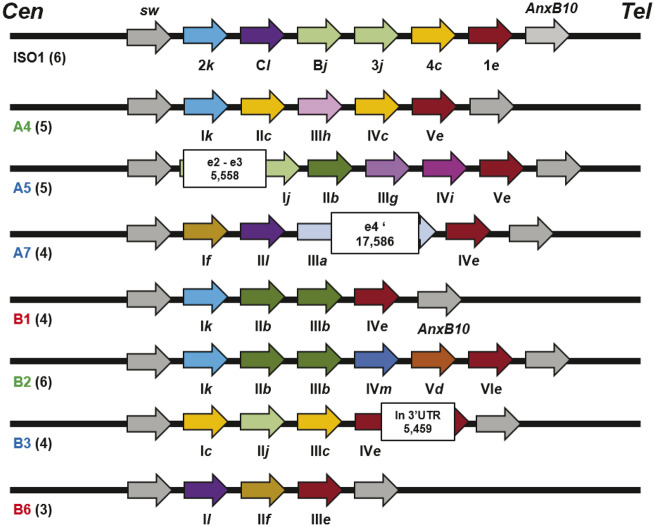
Annotation of the *Sdic* region across seven populations of the DSPR panel. The most reliable organization of the region at 19C1 on the *X* chromosome in the ISO-1 is provided as a reference (Clifton et al. 2017). The region is depicted from centromere (Cen) to telomere (Tel), including the flanking genes *sw* and *AnxB10* (gray-filled arrows). Population names are color-coded based on the broad continental region where they were collected: green, Africa; red, Americas; and blue, Eurasia. The number of annotated *Sdic* copies in reference-quality genome assemblies (Chakraborty et al. 2018, 2019) is indicated in parentheses next to the name of the population. *Sdic* copies in the ISO-1 strain are named as reported (Clifton et al. 2017). In the rest of populations, the copy identifiers are roman numerals according to their relative order from *sw* to *AnxB10*. *Sdic* copies are color-coded, and a lower character (*a–m*) added to their identifier, both indicating the associated paratype. Three TE insertions (solid boxes) are shown, indicating both their size in kb and the location in relation to the gene structure (e, exon). One TE insertion is located within intronic sequence (A5_I), a common occurrence (Chakraborty et al. 2018). In the other two cases, A7_III and B3_IV, the TE disrupts coding and 3′-UTR sequence, respectively. In the first case, the TE has possibly no functional consequence as a premature STOP codon resides upstream of the TE insertion; the apostrophe indicates an ancestral coding exon, which now situates outside of the predicted open reading frame.

Upon annotating the *Sdic* region in these 14 assemblies (fig. 1 and supplementary fig. S1, Supplementary Material online), we found that all assemblies but three (A2, A6, and B4; supplementary text and supplementary fig. S2, Supplementary Material online) show the *Sdic* region unfragmented and flanked by the same genes as in the reference strain, that is, *sw* upstream and *AnxB10* downstream, occupying a proximal position relative to the centromere. All copies of the *Sdic* repeat examined were essentially the same length within and across assemblies. Excluding two unreliable assemblies (A2 and A6) for the *Sdic* region, only those from Cape Town (B2) and Riverside (B4) harbor six copies as in the reference strain (Berlin et al. 2015; Clifton et al. 2017). Overall, we observed a noticeable breadth in CN with a coefficient of variation of 26.8% (*n *=* *12; 4.25 ± 1.14, avg ± SD; 4, median). This CNV contributed to size differences in the *Sdic* region, which ranges from ∼34 kb (Canton-S, A1) to ∼57 kb (Cape Town, B2) (supplementary table S2, Supplementary Material online).

#### CN Estimates from Gene Annotation Are Only Partially Validated

**Fig. 2.**
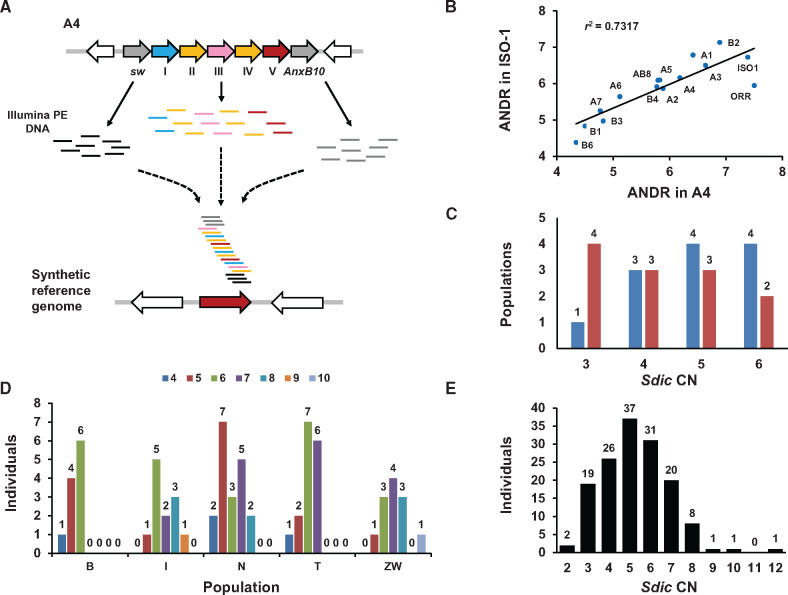
*Sdic* CNV estimation using a read-depth methodology*.* (*A*) Normalized read-depth estimates were obtained using CNVnator (Abyzov et al. 2011). To use as a reference genome, we generated a collection of synthetic *X* chromosomes carrying one *Sdic* repeat each from all the copies in the A4 and ISO-1 strains (only one of them, from the A4 strain, is shown). These synthetic *X* chromosomes also lacked the parental genes *sw* and *AnxB10* (gray-filled arrows), as advised by our benchmarking analysis. Therefore, all Illumina reads belonging to the *Sdic* copies and most of those from the parental genes should presumably map against the *Sdic* copy present in the synthetic genome. Open arrows, genes flanking the *Sdic* region. (*B*) Scatter plot of the averaged normalized read-depth (ANRD) estimates obtained using the synthetic genomes from ISO-1 and A4 for each of the strains assayed. Eliminating the most discordant strain, OR-R, the shown determination coefficient (*r*^2^) becomes 0.901; *r*^2^ is statistically significant (*P < *0.0001) in both cases. These results show that the estimates do not depend on the reference strain used to generate the synthetic reference chromosomes. (*C*) Frequency distribution of populations from the DSPR panel based on the number of structurally distinct alleles in CN that they carry. A2 and A6 are omitted due to obvious errors in the assembly of the *Sdic* region. Blue, CNVnator round-off values; red, gene annotation values. (*D*) *Sdic* CNV across five populations of *Drosophila melanogaster*. Rounded-off average read-depth estimates obtained with CNVnator on the number of *Sdic* copies across 70 strains (each strain represents one individual) are shown (supplementary table S8, Supplementary Material online). The average read-depth estimate is calculated using the values obtained from all synthetic reference *X* chromosomes. Different CNs are color-coded above. CN estimates and sequence coverage were not found to be correlated (*r*^2^*=* 0.0008; *P *=* *0.8198). B, Beijing, *n* = 11; I, Ithaca, *n* = 12; N, The Netherlands, *n* = 19; T, Tasmania, *n* = 16; Z, Zimbabwe, *n* = 12. (*E*) Frequency distribution of all individuals genotyped for *Sdic* CN, that is, OR-R plus the strains from the DSPR and GDL panels, as well as those from a Zambian population.

We attempted to validate the CN estimates obtained from annotating the *Sdic* region in reference-quality assemblies both computationally and experimentally. In the first case, we performed read-depth analyses using CNVnator (Abyzov et al. 2011), which was optimized for the special features of the *Sdic* region (fig. 2*A*; Materials and Methods and Supplementary Text). The final analyses were done using synthetic reference genomes derived from A4 and ISO-1 separately, showing a high degree of agreement between the average read-depth estimates from both analyses (fig. 2*B*). These synthetic genomes contain only one single repeat of *Sdic* and lack the parental genes, removing redundancy across the *Sdic* region*.* Overall, we found a 50% (i.e., seven out of 14 strains) discrepancy rate between the estimates obtained with CNVnator and those from genome annotation (fig. 2*C* andsupplementary tables S2 and S3, Supplementary Material online).

**Fig. 3.**
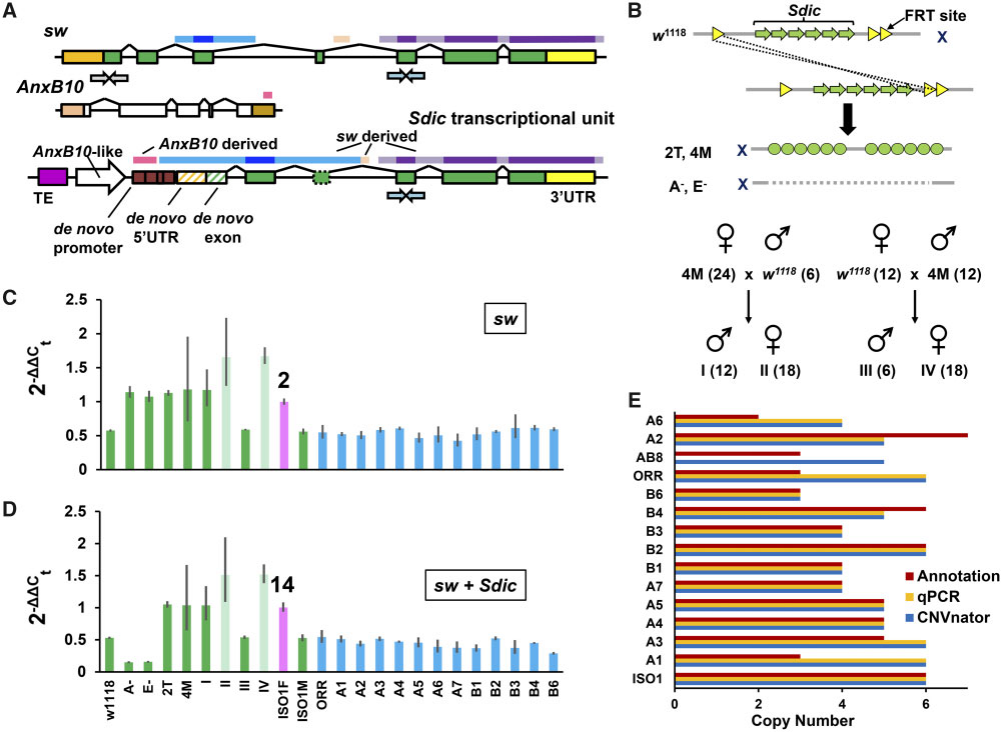
CNV estimates by qPCR. (*A*) Structure of *Sdic* and its parental genes *sw* and *AnxB10.* Colored horizontal bars above the gene models denote those regions donated to the chimeric gene *Sdic* from its parental genes. *Sdic* is part of a repeat also consisting of a partial fragment of the non-LTR retrotransposon Rt1c and an *AnxB10-*like entity, that is, a presumed pseudogene derived from *AnxB10*. *Sdic* exons are shown in green, with the exon one, a de novo exon not translated in *sw*, indicated with green diagonal stripes. A predicted alternatively spliced exon is indicated with a dotted box (Nurminsky et al. 1998). Two sets of primers were designed for the qPCR experiment; one exclusive of *sw* (gray-filled arrows) and the other able to amplify both *sw* and *Sdic* sequence (green-filled arrows)*.* (*B*) Top, *w^1118^*, a strain derived from OR-R (Bingham 1980) and used to generate FRT-bearing strains (Parks et al. 2004), which can be implemented in mating schemes to generate engineered *X* chromosomes carrying the deletion and the duplication of the *Sdic* cluster (middle). These induced chromosomal rearrangements result from FLP-mediated recombination events between FRT sites (see supplementary fig. S3, Supplementary Material online, for further details). Bottom, reciprocal crosses between a strain carrying the wild-type version of the cluster and another carrying its duplication in tandem to obtain progenies with a particular number of *Sdic* copies (in parenthesis). The known CN for *Sdic* and *sw* in each of the synthetic genotypes was used to calibrate our ability to discern differences in CN at the *Sdic* region. (*C* and *D*) Average fold change in CN for the gene *sw* and for *sw* jointly with *Sdic* across a set of control genotypes (green) and across a second set of geographically diverse strains (blue). The difference between the CNs associated with both amplicons corresponds to the number of *Sdic* copies for each genotype. Females from the reference strain (ISOF; pink) were used as calibrator in the estimation of CN. Female genotypes are shown in faint colors. Error bars, SEM. ISOF and ISOM, females and males of the ISO-1 strain; A^−^ and E^−^, deletion-bearing strains; 2T and 4M, duplication-bearing strains; I–IV, genotypes in the progeny from the reciprocal crosses outlined in (*B*). (*E*) Horizontal histogram showing the CN estimates obtained by qPCR, CNVnator, and genome annotation.

We additionally estimated *Sdic* CN using qPCR. Given the structural relationship between *Sdic* and its parental gene *sw*, we estimated *Sdic* CN as the difference between the CN inferred from an amplicon associated with both *sw* and *Sdic*, and another amplicon specific to *sw* (fig. 3*A*; Materials and Methods and supplementary table S4, Supplementary Material online). We first calibrated our ability to discern CN differences across a set of genotypes that correspond with particular strains and their progenies with known CNs for *Sdic* and *sw*. Specifically, we used *w^1118^*, an isogenic strain used to engineer structural variants (Parks et al. 2004), a set of derivative engineered genotypes carrying either the full deletion (Yeh et al. 2012; Clifton et al. 2017) or the duplication in tandem (this work; supplementary fig. S3, Supplementary Material online) of the *Sdic* region, and the progeny from reciprocal matings involving some of these strains (fig. 3*B*). The results strongly supported our ability to correctly infer the number of *Sdic* copies using qPCR assays (Supplementary Text), which were extended to 12 strains belonging to the DSPR panel and OR-R (AB8 was unavailable). In total, 24 genotypes were examined (supplementary table S2, Supplementary Material online and fig. 3*C* and *D*). The comparison of the qPCR and gene annotation estimates showed that they were coincidental for only ∼50% (7/13) of the strains.

Conversely, the comparison of the rounded-off CN values obtained by read-depth analysis estimates and qPCR assays showed a perfect agreement (fig. 3*E* andsupplementary table S5, Supplementary Material online). Using the CNVnator estimates, as they include one more strain than those from qPCR, we noticed that the discrepancies did not follow a consistent trend, that is, CNVnator estimates were in five cases higher and in two cases lower than those from the genome annotation analysis. The three approaches show complete agreement for only seven out of 13 strains investigated (A4, A5, A7, B1, B2, B3, and B6). This, combined with the findings noted above for several assemblies, points to the estimates from the genome annotation analysis as the least reliable. This could presumably result from artifactually collapsing or adding copies while assembling the *Sdic* region, offering a cautionary note to solely depending on reference-quality assemblies when characterizing structural variation in complex regions. Overall, the CNVnator and qPCR estimates confirm that the *Sdic* region has undergone extensive structural remodeling (for CNVnator, *n *=* *14 strains; copies = 4.86 ± 0.95, avg ± SD; CV = 19.54%), harboring four structurally distinct alleles based on CN alone, and showing similar copy range (3–6) across different continental regions (supplementary table S2, Supplementary Material online).

#### SMRT-Based Assembly Properties Affect Accurate Region Recapitulation

To determine what factors affect the inaccurate recapitulation of the *Sdic* region in some assemblies scaffolded with SMRT sequencing reads, we performed a multiple logistic regression to precisely evaluate the predictive power of different assembly metrics when used genome-wide, including sequence coverage, assembly N50 (Earl et al. 2011), and NR50—the median read length above which half of the total coverage is contained (Chakraborty et al. 2018). None of the assembly metrics evaluated turned out to be a good predictor of a faithful recapitulation of the *Sdic* region (supplementary table S6, Supplementary Material online). Subsequently, as assembly metrics fluctuate locally, we focused on the individual reads related to the *Sdic* region, recalculating both coverage and NR50 and adding a few other metrics such as the interpolated size of the region based on CN as estimated with CNVnator. Across strains, the number of reads related to the *Sdic* region was 134 ± 56.8 (avg ± SD), with the maximum and minimum number of reads being 275 (A4) and 53 (A6), respectively (supplementary table S7, Supplementary Material online). We found no strain for which there was at least one sequencing read spanning from *sw* to *AnxB10*. The A4 strain stood out showing the second-highest local NR50 (17.9 kb) and the highest local coverage (∼93×), confirming not only that it is arguably the best assembly of the euchromatin of *D. melanogaster* (Chakraborty et al. 2018) but also in relation to complex regions like *Sdic*. When the metrics were restricted to the *Sdic* region, the multiple logistic regression analysis found that the local coverage has a significant predictive power (*P *=* *0.0057), with a higher local coverage increasing the likelihood of faithfully recapitulating a complex region like *Sdic*. For the seven reliable assemblies within the DSPR panel, the minimum local coverage was ∼29× (B3), with their average coverage being significantly higher than that of the unreliable assembly (∼39× vs. ∼27×, respectively; Kruskal–Wallis, *P *=* *0.015).

### Global Molecular Diversity Patterns in the *Sdic* Region

#### The Sdic Region Is Polymorphic for Structurally Distinct Alleles around the World

Each population included in the DSPR panel and OR-R is derived from a single individual, which prevents an accurate inference of the level of polymorphism and population differentiation, if any, for the *Sdic* region at the structural level. To circumvent this limitation, we used CNVnator on a second panel of isogenic lines, the Global Diversity Lines, derived from five collection sites: Beijing, Ithaca, the Netherlands, Tasmania, and Zimbabwe (Grenier et al. 2015). None of the 70 individuals ultimately considered lacked *Sdic* and 39% featured CNs outside the range seen in the DSPR panel. More importantly, we found up to seven structurally distinct alleles based on variable CN (4–10 copies), with no more than five of these alleles in any given population (minimum = 3; Beijing; maximum = 5, Ithaca, the Netherlands, and Zimbabwe) (fig. 2*D* and supplementary table S8, Supplementary Material online). In all populations, there are at least three structurally distinct alleles at a frequency ≥5%.

Using the *V*_ST_ statistic (Redon et al. 2006), we found that population differentiation in the *Sdic* region is greater than expected by chance alone (*V*_ST_ = 0.1714, *P *=* *0.0023; 10,000 Monte Carlo simulations). Subsequent global and pairwise nonparametric tests showed that the Beijing population features significantly lower CNs than the Zimbabwe and Ithaca populations (supplementary table S9, Supplementary Material online). In fact, the two latter populations exhibit the highest frequencies of structurally distinct alleles carrying the maximum CNs documented (9 and 10). An additional analysis of a third panel of strains from Zambia, each strain corresponding to a different haploid embryo genome, allowed us to zoom in on a different location of *D. melanogaster*’s ancestral distribution range (Lack et al. 2016), extending the detection of additional structural distinct alleles beyond those present in DSPR and GDL individuals; two embryos were found to carry two copies and one with 12 (supplementary fig. S4 and table S8, Supplementary Material online).

#### TE Insertions Contribute to Sdic Structural Variation

We looked for additional structural variants in the assembly of the seven most reliable strains of the DSPR panel for the *Sdic* region. In all strains, the copies are tandemly oriented head-to-tail, consistent with the absence of inversions. Nevertheless, we found three population-specific TE insertions (fig. 1), none of them presumably compromising the protein-coding potential of the copies (supplementary table S10, Supplementary Material online). Considering differences in CN and TE insertions, we find that each population in this subset of strains harbors a structurally distinct version of the *Sdic* region.

#### Sdic Copy Differentiation Affects the Carboxyl End of Sdic Protein Variants

**Fig. 4.**
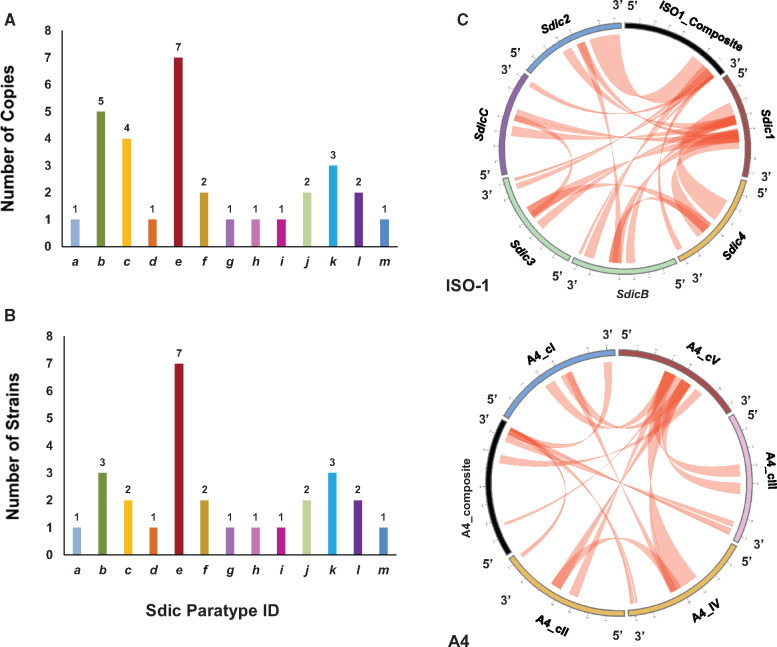
Salient patterns of molecular diversity in the *Sdic* region of seven populations of the DSPR panel. Each of these populations is represented by one isogenic strain derived from one single individual. The different paratypes are color-coded according to figure 1. (*A*) Number of copies in which the 13 Sdic paratypes were present across strains. Each paratype is present as 2.38 ± 1.89 copies, with six of them as a single copy (*a*, *d*, *g*, *h*, *I*, and *m*). (*B*) Presence of the 13 Sdic paratypes across strains. Each strain harbors *Sdic* copies associated with 3–5 paratypes (3.86 ± 0.90; mean±SD), whereas each *Sdic* paratype is present in 1–7 copies across strains (2.17 ± 1.70; mean±SD). For both (*A*) and (*B*), only data from the strains of the DSPR panel considered to be the most reliable for the *Sdic* region were examined. Two additional paratypes are not shown as they are not present in this subset of strains. (*C*) Gene conversion landscape in the *Sdic* region. Circular layout showing the topology of gene conversion events across *Sdic* copies and the composite (in black), that is, the fragments from *sw* plus *AnxB10* that align with *Sdic*. The results from GenConv (Sawyer 1989) are graphed for ISO-1 and A4; equivalent layouts for the other six strains are provided in supplementary fig. S6, Supplementary Material online. Gene conversion was found rampant across strains with an average of 5.6 events per copy and strain, showing distinctive topological patterns. Events involving paratype *e* primarily occur within the interval 2.3–7.2 kb from the start of the repeat, that is, from slightly upstream of the 5′-UTR of the *Sdic* transcriptional unit toward an internal position within the intron between *Sdic*’s exons 2 and 3. In contrast, the events involving *sw* occur 7.2 kb downstream from the start of the repeat, that is, within the intron between *Sdic*’s exons 2 and 3 (supplementary fig. S7, Supplementary Material online).

The most reliable subset of strains harbors 31 *Sdic* copies. Consistent with the age of the region and the occurrence of NAHR and gene conversion events (see below), the level of nucleotide differentiation is very limited among copies both within and across strains (supplementary table S11, Supplementary Material online). This observation holds not only for the *Sdic* transcriptional unit but also for the upstream noncoding interval present at each repeat, including the presumed pseudogene *AnxB10-*like for which we did not find evidence of expression (Materials and Methods; supplementary text and supplementary table S12, Supplementary Material online). Importantly, a given *Sdic* allele can occupy different physical locations within the tandem array across strains and be present as several copies in the same strain. We refer to these *Sdic* alleles as paratypes (Fiddes et al. 2018). Based on particular combinations of diagnostic amino acid motifs spanning ≥5 residues in the presumably encoded products, the copies were categorized into one out of 13 paratypes (*a–m*; fig. 1), adding eight new distinct protein variants to the pool of five previously identified paratypes (Clifton et al. 2017). Like in the ISO-1 strain, the new paratypes show notable differences at the level of length and actual amino acid sequence of the carboxyl-terminus (supplementary table S13, Supplementary Material online), which is due to the preferential location of nucleotide differences in the two exons most proximal to the STOP codon (Clifton et al. 2017). Despite length differences, all copies considered presumably encode proteins with 4–7 WD40 motifs, as seen in the ISO-1 strain (Ma et al. 2019). Further, only one paratype, *e*, is found in all strains, and always present as a single copy and adjacent to the parental gene *AnxB10* (fig. 1). The global paratype diversity generated within the *Sdic* region is reflected in the presence of six paratypes as a single copy in the one strain in which they reside (fig. 4*A*), in the fact that each strain harbors 3–5 paratypes (3.86 ± 0.90; mean±SD; fig. 4*B*), and in that three strains (A5, A7, and B6) carry each resident paratype as a single copy. Overall, the similarity between populations based on CN and paratype composition reflects neither phylogenetic relationship nor geographic proximity (supplementary fig. S5, Supplementary Material online).

#### A Common Landscape of Gene Conversion across Strains

To assess the role of gene conversion in shaping the region’s sequence evolution, and whether its mode of action and magnitude differed among strains, we identified tracts of gene conversion (Sawyer 1989). Gene conversion is rampant across strains, with paratype *e* and *sw* dominating the landscape of events as they contribute to 61% of all detected ones (fig. 4*C* and supplementary fig. S6; table S14, Supplementary Material online). In addition, gene conversion events exhibit common topological patterns along the *Sdic* repeat in all strains, showing a good agreement between boundaries of gene conversion tracts predicted by GeneCov and recombination breakpoints inferred with ACG (O’Fallon 2013) (supplementary figs. S7 and S8, Supplementary Material online).

This gene conversion landscape supports a different chronology for the formation of the *Sdic* multigene family from that proposed based on the ISO1 strain alone (Clifton et al. 2017). In an ancestor of the strains examined, an early *Sdic* copy would have engaged in gene conversion events with the most proximal third of the length of *sw* to its 3′ end. At some point, this early copy duplicated. The paralog adjacent to *sw* continued exchanging DNA tracts with *sw*, whereas the paralog adjacent to *AnxB10* gave rise to paratype *e*. This new cluster configuration likely favored gene conversion between both *Sdic* paralogs, at their 2.3–7.2 kb interval. This, however, limited exchange between *sw* and paratype *e*, possibly owing to their more distant positioning, separated by an intervening copy. Escaping gene conversion events with *sw* permitted paratype *e* to accumulate sequence differences at its 3′ end, a region that evolves under positive selection (Clifton et al. [2017] and below). This scenario is compatible with alternative phylogenetic reconstructions in which all paratype *e* copies from the different strains always conform to a well-supported monophyletic clade, basal to the remaining paratypes (supplementary fig. S9, Supplementary Material online). The branch leading to this clade is comparatively long, despite rampant levels of gene conversion involving paratype *e*, in line with fixed differences at its 3′ region. Additional paratypes would have been formed and eliminated afterward, resulting into a floating set of additional *Sdic* copies, whose divergence would have been confined to sections of the most 3′ third of the *Sdic* transcriptional unit. These additional copies might still be engaged in gene conversion events with the central sequence interval of paratype *e*, limiting further differentiation for that part of the repeat.

#### Positive Selection in Coding and Noncoding Sequences of the Sdic Repeat

The common positional patterns among predicted gene conversion boundaries and recombination breakpoints across the length of the *Sdic* repeat and strains prompted us to assess the impact of positive selection separately for each partition. Overall, we find strong evidence for the action of purifying selection but for the coding fraction of the *Sdic* transcriptional unit, we detect an unequivocal signal of positive selection in subpartition P6.1 (supplementary fig. S8, Supplementary Material online), which encodes part of the carboxyl-termini of the Sdic protein (*P*_adj_= 0.012). In this region, the basal lineage leading to the ancestor of eight nearly identical copies (one copy per strain, corresponding to paratype e), accumulates nonsynonymous changes faster than expected under neutrality. We also identified various lineages in the *Sdic* family tree showing statistical evidence for positive selection in multiple partitions (P1, P3, P5, P6), many of them encompassing noncoding sites (in both internal branches and tips; supplementary table S15, Supplementary Material online). These results are consistent with positive selection playing a major role in driving not only the evolution of the 3′-UTR of the ancestral *Sdic* copy and of the copies that form the diverged clade that corresponds to paratype *e* but also of a fraction of the noncoding sequence elsewhere in the *Sdic* repeat. The 3′-UTRs of the *Sdic* copies in the ISO-1, particularly that of *Sdic1* (paratype *e*), were previously shown to have been extensively remodeled in their miRNA binding site composition relative to *sw* (Clifton et al. 2017).

### *Sdic* Global Expression Level Does Not Correlate with CNV

**Fig. 5.**
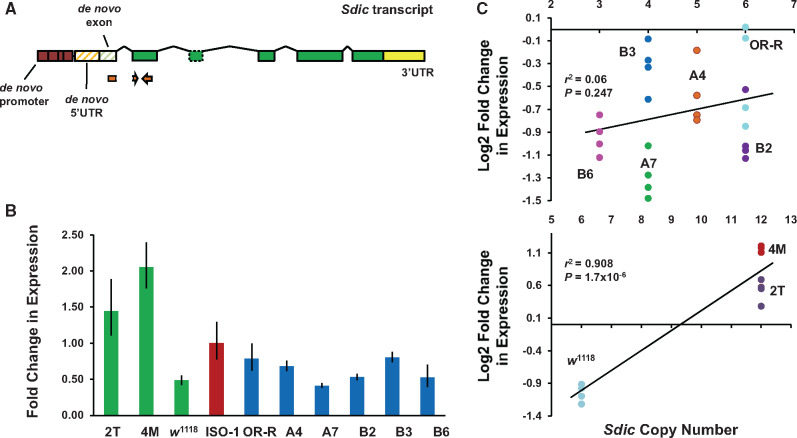
Global expression of the *Sdic* multigene family in whole-body males using qRT–PCR. (*A*) *Sdic* primers are shown relative to the *Sdic* transcriptional unit. See figure 3*A* for details about the relationship of different parts of this transcriptional unit with the structure of the parental genes. Primers were designed upon examining the sequence of all the copies across all the strains of geographically diverse origin, plus ISO-1, making sure that there was no mismatch or gap. The upstream primer was designed spanning the intron between exons 1 and 2 of *Sdic*, with only 5 nt within exon 2, to prevent amplification of *sw*. (*B*) Fold change in expression of ten strains, including ISO-1 (value of 1 on the *y* axis), which was used as calibrator. Green, *w^1118^* and its synthetic derivatives carrying the duplication of the *Sdic* region (2T and 4M). Blue, strains of different geographical origin plus OR-R. Error bars, SEM. (*C*) Linear regression between CN and log2-fold change in expression for the two subsets of strains examined. Each dot represents the values obtained for each biological replicate included in the analysis. Determination coefficients (*r*^2^) and their corresponding *P* values are shown.

Complete gene duplications, that is, those including regulatory sequences, are thought to result in additive changes in transcript abundance that have the potential of affecting organismal fitness (Kondrashov et al. 2002; Kondrashov 2010). To test whether a higher *Sdic* CN actually results in a higher expression level, we estimated the aggregate expression from all *Sdic* copies in males, the sex in which *Sdic* exhibits preferential expression (Clifton et al. 2017). Using qRT–PCR, and with ISO-1 as a reference, we surveyed *Sdic* expression levels across the five strains from the DSPR panel for which there was no discrepancy across methodologies to estimate CN (supplementary table S2, Supplementary Material online) and OR-R, spanning the observed CN range, that is, 3–6 (fig. 5*A* and *B*). Although we found global differences in expression levels (one-way ANOVA, *F* = 9.99, df = 6, *P *<* *0.0001; supplementary table S16, Supplementary Material online), there is limited evidence of significantly different expression across pairwise comparisons mirroring the direction of the differences in CN between strains. Seven of the 21 pairwise comparisons entail a statistically significant alteration in expression (*P *<* *0.05, Tukey–Kramer HSD post hoc test; supplementary table S16, Supplementary Material online), with only four of those comparisons agreeing with the CN differences. For example, strain A7, which harbors four *Sdic* copies, exhibits the lowest *Sdic* expression, being significantly different from B3 (also harboring four copies), A4 (five copies), and OR-R (six copies), but not from B2 (six copies) and B6 (three copies). Relative to the reference strain ISO-1, only three of the six strains surveyed showed significantly different expression (A7, four copies; B2, six copies; and B3, four copies), being lower in all cases. The largest difference in transcript abundance is found between strains with identical CN, B3, and A7 (∼97% more transcript in the former). Overall, we found no evidence of a positive association between transcript abundance and CN in natural populations (*r*^2^= 0.06, *P *>* *0.05; fig. 5*C*).

This substantial decoupling between CN and transcript level could result from buffering mechanisms acting in the face of excessive CN, such as negative feedback loops and access limitations to transcriptional factories in the nucleus (Harewood et al. 2012; Rogers et al. 2017), and from differential composition of expression modifiers acting in *cis-* and *trans-*across populations. To help clarify this extent, we surveyed *Sdic* expression levels in *w^1118^* and its two derivative engineered genotypes carrying a duplication of the *Sdic* region, thus evaluating the impact on gene expression solely resulting from CN differences, without any confounding effect arising from differences in genomic background. Reminiscent of findings with tandemly arranged duplicate pairs of the *D. melanogaster* gene *Adh* (Loehlin and Carroll 2016), we found that duplicating the *Sdic* region in the same genetic background results in statistically significant increases in expression beyond a mere 2-fold change, that is, 100% more: 2T, 158% more; 4M, 209% more (one-way ANOVA, *F* = 61.73, df = 3, *P *<* *0.0001; fig. 5*C* and supplementary table S16, Supplementary Material online). This result suggests that within-strain buffering mechanisms have very little effect on aggregate *Sdic* male expression, and therefore the interplay between *Sdic* CN and expression level in natural populations is primarily shaped by regulatory variants.

### More Functional *Sdic* Copies Do Not Result in Increased Sperm Competitive Ability

When considering the 146 individuals or haploid embryos genotyped for CN using CNVnator, ∼91% of them show within three and seven copies, with decreasing frequencies for CN values outside this range (fig. 2*E*). Given the advantageous effect that *Sdic* confers to males in sperm competition (Yeh et al. 2012), it is not apparent why there are not more individuals carrying higher CNs. Accordingly, we tested whether a substantial increase in CN enhances sperm competitive ability by testing differences for this trait among males carrying the wild-type-like version of this region, its deletion, or its duplication, in all cases in *w^1118^* background.

**Fig. 6.**
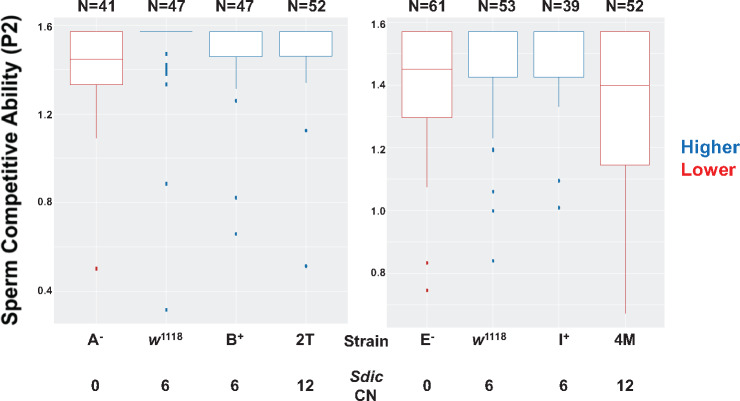
Sperm competitive ability in offense assays for males with different genotypes at the *Sdic* region. Left and right, two strain sets generated in the course of different structural modifications of the *Sdic* region, all of them derived from *w^1118^*. Strains 2T and 4M, *Sdic* duplication-bearing males; A^−^ and E^−^, *Sdic* deletion-bearing males; B^+^, I^+^, and *w^1118^*, wild-type-like presence of the *Sdic* region. The data for 2T and 4M were obtained at the same time as for A^−^, E^−^, B^+^, I^+^, and *w^1118^*; the data for the latter were reported (Yeh et al. 2012). Males from these strains were tested for differences in sperm competitive ability in displacing the sperm from a reference male when they were second to mate in double-mating experiments. The metric to measure sperm competitive ability in this type of experimental setting, P2, informs about the proportion of the progeny sired in double-matings. The angular transformation was applied to the P2 values, which are shown. Box plots show dispersion around the median and are color-coded indicating significantly different sperm competitive abilities (*P*_adj_ < 0.05; supplementary table S17, Supplementary Material online, for the *P* adjusted values from all pairwise contrasts performed). The box plots of male genotypes showing significantly higher sperm competitive ability are shown in blue, whereas those performing poorer are in red. Genotypes with identical color denote no significant differences in the trait assayed. Males from *Sdic* duplication-bearing strains never show higher sperm competitive ability than males carrying the wild-type-like form of the *Sdic* region. In fact, these males can have even lower sperm competitive ability compared with males from *Sdic* deletion-bearing males (4M vs. E^−^). Top, number of females for which their progeny was examined.

In phenotypic tests performed to detect differences in sperm competitive ability between competing males by tracking the fraction of the progeny fathered by different males that have mated with the same female, males carrying the duplication of the *Sdic* region did not exhibit a significantly higher sperm competitive ability (fig. 6). Although there is no perfect consistency in the performance shown by the males of the two duplication-bearing strains, having twice as many copies of *Sdic* as in *w^1118^* decreases sperm competitive ability to the same extent as if no *Sdic* copy is present in the genome (4M vs. E^−^) or does not differ from carrying the default CN in the *w^1118^* background (2T vs. B^+^ and *w^1118^*) (supplementary table S17, Supplementary Material online).

## Discussion

We have generated a detailed portrait of the organization and patterns of intraspecific genetic and functional variation of arguably one of the most recently formed and structurally complex regions in the *D. melanogaster* euchromatin. We find compelling evidence that the *Sdic* region has undergone extensive structural remodeling in natural populations from very diverse geographical origins. Its inherent properties, that is, multiple copies of high sequence identity in the same orientation, and other genomic features can explain the susceptibility of this region to remodeling. For example, close proximity to replication origins has been shown to be related to CNV (Lee et al. 2007; Langley et al. 2012). Interestingly, two origins of replication have been annotated at the 5′ end of *AnxB10* and *sw*, respectively (Eaton et al. 2011). Further, *Sdic* adds to the limited list of NAHR hotspots whose evolutionary dynamics is likely to be influenced by sexual selection, although in this case at the post- rather than premating level (Karn and Laukaitis 2009; Pezer et al. 2015; Pezer et al. 2017).

For a subset of seven cosmopolitan populations from one of the panels analyzed, for which genetic changes could be tracked both at the sequence and structural levels, we found one structurally distinct version of the region per population. This level of variation results from both changes in CN and recent TE insertions. Further, the breadth of CNV was evaluated in six populations from different continents, two of them corresponding to different locations within the presumed ancestral range of *D. melanogaster* (Begun and Aquadro 1993). The extensive degree of CN polymorphism found in these two populations is compatible with a scenario in which the ancestral population that migrated into Eurasia from Africa ∼10,000 years ago (Li and Stephan 2006; Stephan and Li 2007) was polymorphic for *Sdic* CN. Additionally, we observed that many of the structurally distinct alleles based on CN are shared across the populations from the GDL panel, although there is evidence of statistically significant population differentiation involving the Zimbabwe and Beijing populations. This last pattern mirrors previous inferences based on genome-wide SNP data analysis (Grenier et al. 2015).

The frequency distribution for *Sdic* CN in natural populations is far from that expected under a runaway amplification process in which additional functional copies would be correlated with higher expression, ultimately having a directional effect on the phenotype (Brown et al. 1998; Schmidt et al. 2010; Soh et al. 2014). In contrast, we found that intermediate CN values are prevalent, that differences in the aggregate transcript abundance are not correlated with CNV in a geographically diverse set of strains, and that significantly increased *Sdic* expression as a result of artificially doubling CN does not result in enhanced sperm competitive ability based on progeny contribution in double-mating assays. The prevalence of individuals bearing intermediate CN values could result from a scenario of stabilizing selection, or from a mutation-drift equilibrium coupled with the action of purifying selection sculpting the range boundaries as proposed for some multigene families in mammals (Hollox 2008; Teitz et al. 2018).

In relation to *Sdic* expression levels, the lack of correlation between CN and transcript abundance is in line with previous reports in other *Drosophila* species, rat, and in peach-potato aphids (Field et al. 1999; Guryev et al. 2008; Rogers et al. 2017), but it is at odds with a general trend previously reported in *D. melanogaster* (Cardoso-Moreira et al. 2016). At least in relation to the upper end of transcription, buffering mechanisms do not seem to be a good explanation as shown by the enhanced expression documented in our engineered duplications of the *Sdic* region. Alternatively, expression modifiers present in different genomic backgrounds could explain the lack of correlation documented. Such modifiers include regulatory variants in *cis* and *trans* (Lemos et al. 2008; Catalan et al. 2016), as well as alterations of copy functionality by TE insertions or premature termination codons that activate the nonsense-mediated decay pathway (Hug et al. 2016; Scott et al. 2016). Based on sequence analyses in the strains examined, we do not observe overt mutations that could damage promoter activity nor evidence of disruptive mutations that could compromise transcript stability in the reliably annotated *Sdic* copies. Overall, our results suggest that the across-population variation in aggregate male gene expression level for the *Sdic* multigene family is not as much influenced by CN as by population differences in regulatory input, possibly in *trans.*

As for the lack of association between enhanced *Sdic* expression through increased CN and sperm competitive ability, it is not immediately apparent what is the cause. First, the boosting effect of *Sdic* on sperm competitive ability (Yeh et al. 2012) might plateau beyond an unknown threshold expression level. Second, an increased CN might result in enhanced sperm competitive ability, but this beneficial effect is offset by detrimental effects that reduce the viability of the progeny carrying the duplication of *Sdic.* This second scenario is feasible as in the double-mating assays performed, differential sperm competitive ability is inferred through differential progeny contribution between competing males carrying different CN when they are second to mate (P2) rather than by a more reliable method based on the direct observation of the sperm from those genotypically different males in the female reproductive tract (Jayaswal et al. 2018). This would result in no significantly different P2 values between males carrying 6 and 12 *Sdic* copies even though there were true differences in sperm displacement (Civetta and Ranz 2019). Further, reduced progeny viability can be related to increased expression above a threshold, which is conceivable in the case of *Sdic* as it is expressed in somatic tissues of both genders, having the potential to affect other traits beyond sperm competition (Clifton et al. 2017). The nature of this detrimental effect could take place directly by triggering molecular imbalance, energetic waste, or titrating out limiting factors such as RNA polymerases and ribosomes (Rice and McLysaght 2017), or indirectly through an excessive downregulation of the parental and dosage-dependent gene *sw*, as *Sdic* can presumably compete with it in the context of the interactions that *sw* establishes with several protein complexes (Boylan et al. 2000; Boylan and Hays 2002). Alternatively, a putatively reduced progeny viability might be unrelated to an increased expression and instead be linked to an enhanced genome instability with higher CN (Didion et al. 2015; Fouche et al. 2018). More refined assays and functional tests should help support or refute these possibilities. At this point, we are only certain of a boosting effect on sperm competitive ability when *Sdic* is expressed in males with six copies relative to males lacking *Sdic* (Yeh et al. 2012), an effect that is not detectable when this CN doubles. Only by testing additional intermediate CN values it will be clearer the fitness-dosage interplay in the case of *Sdic* (Kondrashov 2010).

In contrast to the relatively constrained range of CN and lack of correlation between transcript abundance and CN in natural populations, the *Sdic* region shows a remarkable capability to generate protein diversity in each strain that could be reliably analyzed. We found extensive paratype breadth primarily associated with distinct 3′ carboxyl ends, no evidence of a particular paratype being preeminent in CN within any given strain, and only one of the 13 paratypes—paratype *e*—being present in all strains. This paratype shows strong evidence of having evolved under positive selection both at coding and noncoding levels. Further, this paratype diversity has accumulated despite profuse gene conversion events. The topology of the gene conversion landscape shows extensive commonalities across strains, with the fixed paratype *e* and the parental gene *sw* being major mutually exclusive contributors along the *Sdic* repeat. As these patterns have been documented in cosmopolitan strains, it will be interesting to determine whether they hold in strains from the ancestral range of *D. melanogaster*.

Collectively, our results suggest that *Sdic* CNV in contemporary populations of *D. melanogaster* secures a minimal necessary expression level across different genomic backgrounds and sexual selection regimes, serving also as a substrate to prevent nucleotide change via gene conversion and NAHR events for essentially all the *Sdic* repeat but the two most 3′ exons and the 3′-UTR of *Sdic* copies (Rozen et al. 2003; Teitz et al. 2018). Equally important, maintaining multiple copies that encode different and possibly fully functional paratypes is compatible with a mechanism that safeguards functional diversity at the protein level (Traherne et al. 2010) while enabling expression profile diversification. *Sdic* copies in conventional laboratory strains show evidence of expression divergence across life stages and anatomical parts of the adult (Clifton et al. 2017), which is concurrent with profound 3′-UTR remodeling. At least for the copies associated with paratype *e*, we find evidence of positive selection acting on this portion of the *Sdic* repeat. An equivalent pattern could be taking place for copies of the same paratype but in different populations. Functional characterization of a set of strains with different CN and paratype composition can be highly informative relative to the extent of evolutionary tinkering, that is, the magnitude and mode of diversification of expression attributes, as well as to precisely evaluate the role of putative disruptive mutational events such as TEs during the early stages of formation and consolidation of *Sdic* and similar tandemly repeated multigene families in eukaryotic genomes.

## Materials and Methods

### Fly Husbandry

A combination of strains, including some with wild-type genotypes of diverse geographical origin (King, Macdonald, et al. 2012) and others carrying synthetic genotypes, was used (supplementary table S1, Supplementary Material online). Flies were reared on dextrose–cornmeal–yeast medium in a 25 C chamber under constant lighting conditions.

### Engineering the Duplication of the *Sdic* Region

Engineered duplications of the *Sdic* region were generated using TE-bearing strains with *w^1118^* genomic background (supplementary table S1, Supplementary Material online) (Parks et al. 2004), and following the same mating scheme used previously for deleting the region (supplementary fig. S3*A*, Supplementary Material online) (Yeh et al. 2012). Validation of the engineered duplications was done by inspecting eye color of particular male progeny and by performing a set of diagnostic PCR controls (supplementary fig. S3*B*, Supplementary Material online). See supplementary table S4, Supplementary Material online, for the primers utilized.

### Sperm Competition Assays

Offense double-mating experiments for duplication-bearing males were performed as reported (Yeh et al. 2013), and concomitantly with those for other male genotypes whose results were already published (Yeh et al. 2012). Briefly, sperm competitive ability for any given male genotype was calculated with the P2 metric, which measures the relative contribution of the second male to mate to the total progeny of doubly mated females. The angular transformation was applied to the P2 values (Sokal and Rohlf 1994). Transformed P2 values were stored at Dryad repository (https://doi.org/10.7280/D1RH56).

### In Situ Hybridization

To further assure that the engineered duplication of the *Sdic* region was generated in tandem, in situ hybridization on polytene chromosomes of the strains 2T and 4M was performed as described (Ranz et al. 1997). Probe and signal detection are as reported (Yeh et al. 2012). Further, in order to test the recapitulation of the *Sdic* region in the assembly of the strain A2, in situ hybridization on mitotic chromosomes from larval brains was executed as reported (Pimpinelli et al. 2000). The probe used spans a common region between *Sdic* and *sw.* See supplementary table S4, Supplementary Material online, for the primers utilized to generate the probes.

### Genome Assemblies

Assemblies corresponding to the 13 strains from the Drosophila Synthetic Population Resources (King, Merkes, et al. 2012) plus OR-R were obtained from the NCBI bioproject PRJNA418342. These assemblies were scaffolded with SMRT sequencing reads and polished with Paired End 100 Illumina reads, and are characterized by N50 values ≥ 18.5 Mb (average ∼ 21.2 Mb), coverages for the euchromatic fraction ≥ 36× (average ∼ 70×), and complete BUSCO values ≥ 99.9% (Chakraborty et al. 2018, 2019). The Oxford_Nanopore- and Bionano-based assemblies (Solares et al. 2018) were obtained from https://github.com/danrdanny/Nanopore_ISO1 (last accessed February 1, 2019) and the Nanopore sequencing reads retrieved from the NCBI bioproject PRJNA433573.

### *Sdic* Region Annotation

We used BlastN (Altschul et al. 1990) to locate the 5′ section of *sw* and the 3′ section of *AnxB10* to identify the boundaries of the *Sdic* region in each genome assembly. To extract the region from these assemblies, we used SAMtools/1.3 (Li et al. 2009) using the coordinates from BlastN plus 10 kb added to each side. Annotation of the *Sdic* region was done by searching for sequence motifs corresponding to exon 1 as in the ISO-1 assembly (Clifton et al. 2017). *Sdic* copies were numbered sequentially from *sw* to *AnxB10.* Raw reads associated with the *Sdic* region in each assembly were retrieved for detailed analyses upon identification using BlastN and mapped against the corresponding assembly using minimap2 (Li 2018). Additional features, essentially TE insertions, were characterized by BlastN through FlyBase (dos Santos et al. 2015), and their junctions confirmed by PCR; see supplementary table S4, Supplementary Material online, for the primers utilized. Open reading frames were inspected in MEGA X (Kumar et al. 2018), and the number of WD40 motifs associated with each putatively encoded Sdic protein determined according to a specialized database for WD40-repeat proteins (Ma et al. 2019).

### Read-Depth Analysis

CNVnator (Abyzov et al. 2011) was used to survey CNV in the *Sdic* region using the “-genome” option and a bin size of 100 nt. Illumina sequencing outputs for the DSPR panel (King, Merkes, et al. 2012) and the ISO-1 strain (Langley et al. 2012) were retrieved from GenBank and mapped against a collection of synthetic reference genomes. These synthetic genomes were derived from the assemblies of the A4 and ISO-1 strains. Each synthetic genome contains a different single *Sdic* copy of those present in the mentioned assemblies and lacks the parental flanking genes *sw* and *AnxB10* (Supplementary Text). For any given strain surveyed, the average among all the read-depth estimates obtained from the different reference assemblies was calculated and then rounded off to its closest integer. From this value, 1 was subsequently subtracted because of the contribution of reads from the flanking genes *sw* and *AnxB10* to the read-depth estimates as, combined, they behave essentially as an additional *Sdic* copy. Given the overall high agreement between the average read-depth values obtained using the reference genomes derived from A4 and ISO-1 (Supplementary Text), only those from A4 were used in subsequent surveys of CNV across two additional panels of strains: PRJNA268111 (Grenier et al. 2015); and SRP006733 (Lack et al. 2016). As for these two additional panels of strains no qPCR estimates were available, we adopted the conservative criterion of considering read-depth average values from those strains showing CNV target sizes within reasonable boundaries, that is, 7.2–8.0 kb; in A4, *Sdic* copies range in size from 7.4 to 7.75 kb. Read-depth estimates associated with reference genomes for which the CNV target size was outside of the indicated range were omitted. Only strains for which the number of reliable read-depth estimates were 4–5 were considered in downstream analyses.

### Population Differentiation

The *V*_ST_ statistic (Redon et al. 2006) was calculated for the CNVnator estimates as *V*_ST_ = (*V*_T_−*V*_S_)/*V*_T_, where *V*_T_ is the total variance in CN among all the considered individuals and *V*_S_ is the average of the variance within each single population, weighted for size. The calculation of the *V*_ST_ statistic was done for the rounded-off CN values, the uncorrected average read-depth values, and their log2, finding no difference. The probability of finding *V*_ST_ values equal or higher than that observed given the data was assessed by performing 10,000 simulations of bootstrap resampling.

### qPCR CNV Assays

For each interrogated genotype, three genomic DNA extractions, that is, biological replicates, were performed. In each extraction, 20 entire whole bodies from <10-day posteclosion individuals were homogenized with motorized pestles in 1.5 ml tubes. Genomic DNA was extracted using the Qiagen’s Puregene Core Kit B, and further purified using Zymo Research’s Genomic DNA Clean & Concentrator-10 kit following manufacturer’s instructions. DNA purity was confirmed with a NanoDrop 8000 spectrophotometer (Thermo Fisher), and the specificity of expected amplicons by agarose gel electrophoresis of the qPCR products and the analysis of the melting curves from the qPCR instrument. DNA concentrations were measured using a Qubit 2.0 fluorometer with either Qubit dsDNA BR Assay Kit or Qubit dsDNA HS Assay Kit reagents when appropriate. Real-time qPCR CNV assays were performed accommodating *Sdic*’s chimeric nature, which prevents designing reliable *Sdic*-specific primers. Thus, the number of *Sdic* copies was inferred by performing two sets of qPCR assays in which the first set was specific to s*w* whereas the second annealed with both *sw* and all *Sdic* copies (*Sdic/sw*). Accordingly, the number of *Sdic* copies in any given genotype was inferred by subtracting the number of *sw* copies from the number of *Sdic/sw* copies. Raw CNs estimates were obtained accounting for variable primer efficiencies for the gene of interest and the reference gene (Pfaffl 2001). A randomly chosen single copy autosomal gene *Triose phosphate isomerase* (*Tpi*) was used as a reference. Real-time PCR experiments were performed in 20 µl reactions using PowerUP SYBR Green Master Mix (Applied Biosystems), 5 µM of each primer, and ∼30 ng of purified genomic DNA in 96-well plates on a Bio-Rad CFX-96 1000 touch real-time PCR instrument. Primer sets are listed in supplementary table S4, Supplementary Material online. The average raw gene CN across genotypes was calculated relative to ISO-1 females. Calling CN was done by rounding average raw CN estimates to the nearest integer. Original Ct values were stored at Dryad repository (https://doi.org/10.7280/D11091).

### qRT–PCR Expression Assays

Experiments were done using four replicates of total RNA extractions from whole-body males with a CFX-96 1000 touch real-time instrument (BioRad) using the PowerUP SYBR Green Master Mix (Applied Biosystems) with 1 µl cDNA in a 20 µl reaction. Total RNA was extracted from ten strains (fig. 5) using TRIzol reagent (Thermo Fisher) following manufacturer instructions. Fifty naive males per replicate per strain were systematically sacrificed at 3 pm to control for circadian rhythms and extracted on separate days to avoid strain cross-contamination. DNA traces were subsequently eliminated using the RNeasy mini kit with DNase I (Qiagen). RNA integrity, purity, and concentration were assessed using gel electrophoresis, Nanodrop, and a Qubit RNA BR assay kit, respectively. Each sample was converted to cDNA using 1.5 µg total RNA and the SuperScript IV first-strand synthesis system with an RNase inhibitor (Invitrogen). Effective reverse transcriptase reactions were confirmed through successful RT–PCR of the gene *Gapdh2*. The gene *clot* was used as the reference gene and males from ISO-1 were used for calibration. Expression estimates were obtained accounting for variable primer efficiencies for the gene of interest (*Sdic*) and the reference gene (Pfaffl 2001). Primers used are provided in supplementary table S4, Supplementary Material online. Primer design for *Sdic* took into consideration sequence differences with *sw* and *AnxB10* to confidently survey solely *Sdic* expression, as well as perfect sequence conservation across copies and strains to prevent any copy or population bias. Original Ct values were stored at Dryad repository (https://doi.org/10.7280/D1W98H).

### Expression Profiling of *AnxB10*-Like

Thirty-eight libraries representing 29 biological conditions throughout the *D. melanogaster* life cycle (Graveley et al. 2011) were downloaded from the NCBI FTP site (supplementary table S12, Supplementary Material online). Reads with remaining adapters or with a quality value *Q *≤* *20 were discarded. All remaining reads were then examined for >70-nt alignments with a 130-nt sequence that includes a core motif distinctive of three of the *AnxB10*-like copies (ATAGGTCAGTATATA*CATA*TTTAACTGTTCCGTT; underlined, insertion absent in *AnxB10*) using an in-home script that incorporated the local alignment function from the Biopython package (Cock et al. 2009). The whole core motif was required to be part of the alignment with no mismatch or gap allowed; the extension of the alignment upstream or downstream could contain a single-nucleotide mismatch or indel. An in-house Python script was used to ultimately determine the number of sequencing reads fulfilling the above conditions.

### Gene Conversion Analysis

Multiple sequence alignments (MSA) for the *Sdic* repeats in each strain and for all strains for which their genome assemblies were dubbed as reliable were generated and aligned with MUSCLE within MEGA X (Kumar et al. 2018). Each MSA included a synthetic composite sequence consisted of *Sdic*’s equivalent regions in *sw* and *AnxB10*. Levels of nucleotide differentiation were calculated under a Jukes–Cantor substitution model in MEGA X. All positions containing gaps and missing data were eliminated (completed deletion option). Gene conversion tracts were inferred using the GeneConv software (Sawyer 1989) under the assumption that no nucleotide mismatch occurred among the tracts, thus limiting the number of false positives. In addition, only gene conversion tracts with an associated probability < 0.05 after correcting for multiple tests were considered. Inference of recombination breakpoints was done with the ACG software (O’Fallon 2013) under 20,000,000 iterations and a burn-in period of 5,000,000. Circular layouts showing the topology of gene conversion events in each strain were generated with the Circos software (Krzywinski et al. 2009).

### Phylogenetic Analysis of the DSPR Strains

Contigs containing the mitochondrial genome of each DSPR strain and OR-R were identified via BlastN and extracted from genome assemblies using SAMtools/1.3 (Li et al. 2009). The mitochondrial genome sequence from the reference ISO-1 strain was retrieved from GenBank (accession number: KJ947872) and included in the analysis. Sequence alignment was generated using MUSCLE and subsequently minimally curated by visual inspection. The best model of nucleotide evolution was found to be the Hasegawa–Kishino–Yano model (Hasegawa et al. 1985). The evolutionary history was inferred by using the Maximum Likelihood method. Initial tree(s) for the heuristic search were obtained automatically by applying Neighbor-Joining (NJ) and BioNJ algorithms to a matrix of pairwise distances estimated using the Maximum Composite Likelihood (MCL) approach, and then selecting the topology with superior log likelihood value. A discrete Gamma distribution was used to model evolutionary rate differences among sites (5 categories; +*G*, parameter = 0.0500). The rate variation model allowed for some sites to be evolutionarily invariable ([+*I*], 49.13% sites). All positions containing gaps and missing data were eliminated (complete deletion option). The final data set included 17,964 nucleotide sites. Bootstrapping (1,000 replicates) was performed to determine the confidence of the branches (Felsenstein 1985). Evolutionary analyses were conducted in MEGA X (Kumar et al. 2018).

### Phylogenetic Analysis of Annotated *Sdic* Copies

The phylogenetic relationship among the *Sdic* copies from a subset of strains from the DSPR panel was inferred using a MSA including all *Sdic* copies and composites, and RAxML 8.1.2 (Stamatakis 2014), under a GTRGamma model of sequence evolution. The resulting topology was evaluated through 1,000 bootstrap replicates. This topology is very similar to an alternative one as inferred with PhyML 3.0 (Guindon et al. 2010), which is based on the best-fit substitution model HKY85+G + I with four gamma categories according to SMS (http://www.atgc-montpellier.fr/sms/; last accessed October 21, 2019).

### Positive Selection Analysis

The software package HyPhy (Kosakovsky Pond et al. 2020) was used to test for positive selection acting on coding and noncoding *Sdic* sequences. The adaptive branch-site random effects model (aBSREL; Smith et al. [2015]) and the batch script written by Oliver Fredigo (Haygood et al. [2007]; upgraded to run on Hyphy version 2.5, https://github.com/spond/TestForPositiveSelection/nonCodingSelection.bf; last accessed October 21, 2019) were applied to the coding and noncoding regions, respectively, of the MSA of the *Sdic* repeat in all strains, including the synthetic composite sequences from different strains, and the composite sequence consisted of their corresponding orthologous stretches to *sw* and *AnxB10* in *D. simulans*, which was used as a more external outgroup. See Supplementary Materials for further details. To accommodate for the different gene tree topologies and total branch lengths of sampled genealogies for each partition (or subpartitions) along the MSA identified by the ACG recombination breakpoints, we conducted the test separately for each of these partitions using their respective gene tree (one per partition).

### Statistical Analyses

One-way ANOVA and post hoc Tukey’s HSD tests for detecting differences in mRNA levels across genotypes were done in JMP 12.2.0 (SAS Institute Inc.). Nonparametric Kruskal–Wallis H and pairwise Stell–Dwass tests, which corrects for multiple testing, for detecting differences in sperm competitive ability among genotypes as well as for assessing differences in CN among populations from the GDL panel were done also with the same statistical package. Bootstrap resampling, hierarchical clustering, and logistic regression analyses were done in R (R Development Core Team 2016). 

## Supplementary Material

Supplementary data are available at *Molecular Biology and Evolution* online.

## Supplementary Material

msaa109_Supplementary_Data
