# Supplementary material for: Understanding the Early Evolutionary Stages of a Tandem Drosophilamelanogaster-Specific Gene Family: A Structural and Functional Population Study
Source: Mol Biol Evol. 2020 May 2;37(9):2584–600. doi: 10.1093/molbev/msaa109 (PMC7475035; doi:10.1093/molbev/msaa109)
Supplement: msaa109_Supplementary_Data [file msaa109_supplementary_data.pdf]

SUPPLEMENTARY MATERIALS

Understanding the early evolutionary stages of a tandem *Drosophila melanogaster*-specific gene family: a structural and functional population study

Bryan D. Clifton,[1] Jamie Jimenez,[1] Ashlyn Kimura,[1] Zeinab Chahine,[1] Pablo Librado,[2] Alejandro Sánchez-Gracia,[3,4] Mashya Abbassi,[1] Francisco Carranza,[1] Carolus Chan,[1] Marcella Marchetti,[5,6] Wanting Zhang,[7] Mijuan Shi,[7] Christine Vu,[1] Shudan Yeh,[†,1] Laura Fanti,[5,6] Xiao-Qin Xia,[7] Julio Rozas,[3,4] and José M. Ranz*[,1]

[1]Department of Ecology and Evolutionary Biology, University of California Irvine, Irvine, CA

[2]Laboratoire AMIS CNRS UMR 5288, Faculté de Médicine de Purpan, Université Paul Sabatier, Toulouse, France

[3]Departament de Genètica, Microbiologia i Estadistica, Universitat de Barcelona, Barcelona, Spain

[4]Institut de Recerca de la Biodiversitat, Universitat de Barcelona, Barcelona, Spain

[5]Istituto Pasteur Italia, Fondazione Cenci-Bolognetti, Rome, Italy

[6]Department of Biology and Biotechnology “C. Darwin”, Sapienza University of Rome, Rome, Italy

[7]Institute of Hydrobiology, Chinese Academy of Sciences, Wuhan, Hubei Province, China

†Present address: Department of Life Sciences, National Central University, Taoyuan City, Zhongli District, Taiwan

[*]**Corresponding author:** E-mail: jranz@uci.edu

Supplementary Text

Organization of the *Sdic* region in the reference strain of *D. melanogaster*

The structural and sequence features of the *Sdic* region in the ISO-1 reference strain have been subject to recurrent updates in different releases (Ranz and Clifton 2019). A comparison across assemblies (Clifton, et al. 2017), including Release 6 (dos Santos, et al. 2015) and others generated with long sequencing reads, pointed to one scaffolded with single-molecule real-time (SMRT) (Kim, et al. 2014) sequencing reads as the most accurate: GCA_000778455 or *Berlin* hereafter (Berlin, et al. 2015). This reconstruction of the *Sdic* region entails discrepancies in copy number (six instead of seven) and internal positioning within the array in relation to Release 6. Further support for this different reconstruction derives from an independent assembly that used the same SMRT sequencing input, Illumina sequencing reads (Langley, et al. 2012), and a different computational pipeline (Chakraborty, et al. 2016). The nucleotide-to-nucleotide comparison of the *Sdic* region between these two assemblies uncovered no discrepancy relative to copy number, orientation, or internal positioning, displaying just 9 nt differences, 7 of them part of nucleotide runs.

To further test the reliability of our CN estimate for the *Sdic* region in the ISO-1 strain independently from SMRT-based assemblies, we adopted two strategies. First, we examined an Oxford_Nanopore assembly finding five copies, and another assembly using Bionano Irys finding three copies (Solares, et al. 2018). In the case of the Nanopore assembly, up close examination of 112 sequencing reads associated with the *Sdic* region found no evidence of any of them spanning the whole region (from *AnxB10* to *sw*), providing no convincing indication that the recapitulation of the *Sdic* region was done reliably. Additionally, we performed a read-depth analysis using CNVnator (Abyzov, et al. 2011), finding a normalized read depth compatible with 6 copies (see below and Material and Methods). Collectively, we concluded that the *Berlin* assembly should be used as a reference for the *Sdic* region.

Assemblies with a fragmented *Sdic* region

In three assemblies of the DSPR panel (A2, A6, and B4), we found the *Sdic* region fragmented. Fragmentation was associated with the presence of assembly gaps, which were not supported by further scrutiny of individual SMRT reads associated with the *Sdic* region as we found reads that precisely recover the stretches that presumably correspond to the assembly gaps. In the case of A2, and in addition to examining the reads associated with this region, we performed *in*

situ hybridization on mitotic chromosomes finding a single signal, which indicated that the clustering of the *Sdic* copies at two different sites of the *X* chromosome is an assembly artifact (Fig. S2).

Benchmarking of CNVnator

First, we examined under which conditions CNVnator (Abyzov, et al. 2011) can provide reliable CN estimates given the complexity of the *Sdic* region, *i.e.* the presence of multiple copies with high sequence identity among themselves, as well as with their flanking single-copy parental genes, *AnxB10* and *sw*. To this end, we used arguably the most reliable assemblies so far generated in *D. melanogaster*: GCA_000778455 (Berlin, et al. 2015) for ISO-1; and GCA_002300595.1 for A4 (Chakraborty, et al. 2018). First, we generated a set of synthetic *X* chromosomes for the A4 and the ISO-1 strains in which different *ad hoc* modifications were implemented, *i.e.* deleting all but one *Sdic* copy and the parental genes. A separate synthetic *X* chromosome was generated for each *Sdic* copy in both strains, five from A4 and six from ISO-1, which were used as references for read-depth analysis. The average read-depth values obtained with the sequencing data of the A4 strain were 6.18 and 6.16 when using the A4 and the ISO-1 synthetic reference chromosomes, respectively. Rounding off the average between both values to the closest integer and subtracting one because of the contribution of the reads from the parental genes, the estimated number of *Sdic* copies in A4 is 5. Following the same rationale with the ISO-1 strain, the estimated number of copies was 6 (average read-depth values were 7.38 and 6.73 when using the A4 and the ISO-1 synthetic reference chromosomes, respectively). These estimated numbers are identical to the number of copies found by annotating the indicated assemblies. For 13 strains of the Drosophila Synthetic Population Resources (King, et al. 2012b) and OR-R, the average read depth values across the five and six reference genomes from A4 and ISO-1, respectively, were highly correlated ($r^2 = 0.73$, $P < 0.0001$; Fig. 2B; Table S3). Further, we also examined whether sequence coverage could be positively correlated with CN estimates, finding no evidence. Specifically, we parsed this association in two sets of strains, with the first including 70 datasets from the Global Diversity Lines (Grenier, et al. 2015), and the second including 63 datasets from a Zambian population (Lack, et al. 2016a). For the first set, $r^2 = 0.0008$ ($P = 0.8198$) and for the second $r^2 = 0.0055$ ($P = 0.5661$).

Calibration of qPCR assays

Our control experiments with *sw* confirmed our ability to discern between 1, 2, and 3 copies (Table S2; Fig. 3C). This variation in copy number for *sw* is associated with differences between males and females of the ISO-1 strain, males carrying 2 copies of endogenous *sw* as a result of an induced duplication of the region (2T and 4M; this work), males carrying 2 copies of a *sw* transgene on chromosome 2 upon making it homozygous (A^- and E^-; (Clifton, et al. 2017), and heterozygous females possessing 3 copies as a result of carrying one chromosome with the wildtype configuration for the *Sdic* region and another chromosome with its duplicated version (II and IV in Fig. 3B). For these same genotypes, the estimates about the number of copies of *Sdic* were also identical to the expectation (Fig. 3D): 6 and 12 copies for the males and females of the ISO-1 strain, respectively; 0 copies for the males that carry the deletion of the *Sdic* region (A^- and E^-; (Clifton, et al. 2017)); 12 copies for the males that carry the duplication of the *Sdic* region (4M; this work); and 6, 12, or 18 copies in particular progenies from controlled crosses involving w^{1118}, 2T, and 4M (I-IV in Fig. 3B). The only exception to this good agreement was the estimate for the males from the duplication strain 2T, for which the qPCR estimate was of 12.5 copies instead of 12. Collectively, these results are consistent with a suitable ability to infer the number of *Sdic* copies through our qPCR assay at least between 0 and 18.

Patterns of nucleotide variation across the *Sdic* repeat

For the fraction of each *Sdic* repeat that corresponds to the *Sdic* transcriptional unit, the magnitude of within-strain pairwise sequence identity at the nucleotide level was very similar across the strains considered, with median sequence identity values ranging from 98.62% (B3) to 99.53% (B2); 99.44% when all 31 copies are considered jointly (Table S11). Nevertheless, nucleotide differences in the two exons most proximal to the *Sdic* stop codon result in notable differences at the amino acid level, impacting the length of the putatively encoded variants as previously documented in the ISO-1 strain (Clifton, et al. 2017). These variants varied by up to 29% in length (388-544 residues; Table S13). Further, and also within the *Sdic* transcriptional unit, there are 112 nt corresponding to the presumed *Sdic* promoter (Nurminsky, et al. 1998). We found two additional promoter sequences in relation to the two previously documented (Clifton, et al. 2017). Both additional promoters show nucleotide differences at the same two sites already known to vary among previously delineated promoter sequences of *Sdic* (Clifton, et al. 2017).

We also examined the level of nucleotide differentiation at other sequence intervals that are part of the *Sdic* repeat, *i.e.* ~1,800 nt corresponding to a combination of non-deleted intervals of the canonical sequence of the TE *Rt1c*, and a ~850 nt portion corresponding to the presumed pseudogene *AnxB10*-like (Fig. 3A). We found a striking degree of conservation (number of base differences per site assuming a Jukes-Cantor substitution model; TE *Rt1c*, d = 0.005; *AnxB10*-like, d = 0.002; *Sdic* exonic sequence, d = 0.007). For *AnxB10*-like, we examined the possibility that this strong nucleotide conservation could actually reflect functional constraints contrary to previous reports (Yeh, et al. 2012a). By using an in-home pipeline that tracks small sequence motifs to differentiate expression between very similar duplicated sequences (Clifton, et al. 2017), we screened RNA-seq datasets corresponding to 29 biological conditions (Material and Methods; Table S12), finding no evidence of *AnxB10*-like expression. In the absence of evidence for functionality, the high-level sequence conservation for these intervals of the *Sdic* repeat might be suggestive of structural constraints.

Detecting positive selection across the *Sdic* repeat

Several approaches were used to determine the pattern of sequence evolution across the *Sdic* repeat taking into account the presence of both coding and noncoding sequences. The first method was used to test if positive selection occurred on *Sdic* protein-coding sequences (*i.e*. whether there is proportion of sites with an excess of nonsynonymous substitutions in relation to the expectation under a neutral model) for each branch of the phylogeny. In this model, the number of site classes with a particular nonsynonymous to synonymous rate ratio (ω) in each branch is not fixed but estimated using a small sample AIC. Then, a likelihood-ratio test (LRT) was used to compare the positive selection to the null model (classes with $\omega > 1$ are not allowed), and the *p*-value for each branch was corrected for multiple testing using the Holm-Bonferroni correction (Holm 1979). Similarly, the batch script for detecting positive selection on noncoding sites evaluates whether the substitution rate in this class of sites exceeds significantly a neutral class of sites (here represented by the synonymous sites). In this case, under the null model, the number of noncoding site classes for each branch is set to three: (i) those that are selectively neutral; (ii) those evolving under purifying selection; and (iii) those completely constrained in background lineages (BG) or neutrally evolving in foreground lineages (FG). In the alternate model, this third class of sites is forced to evolve under positive selection in the foreground lineages, and an extra class of sites, neutrally evolving in BG and positively selected in FG, is added. Thus, under this configuration, the relaxation of purifying selection at

some sites is already accounted for by the null model. The LRT was used to compare these two nested models by setting each of the branches of the *Sdic* tree (reconstructed using RAxML and MSA positions) as a background lineage in an independent test. Final *p*-values were adjusted for multiple comparisons using the False Discovery Rate (FDR) correction (Benjamini and Hochberg 1995).

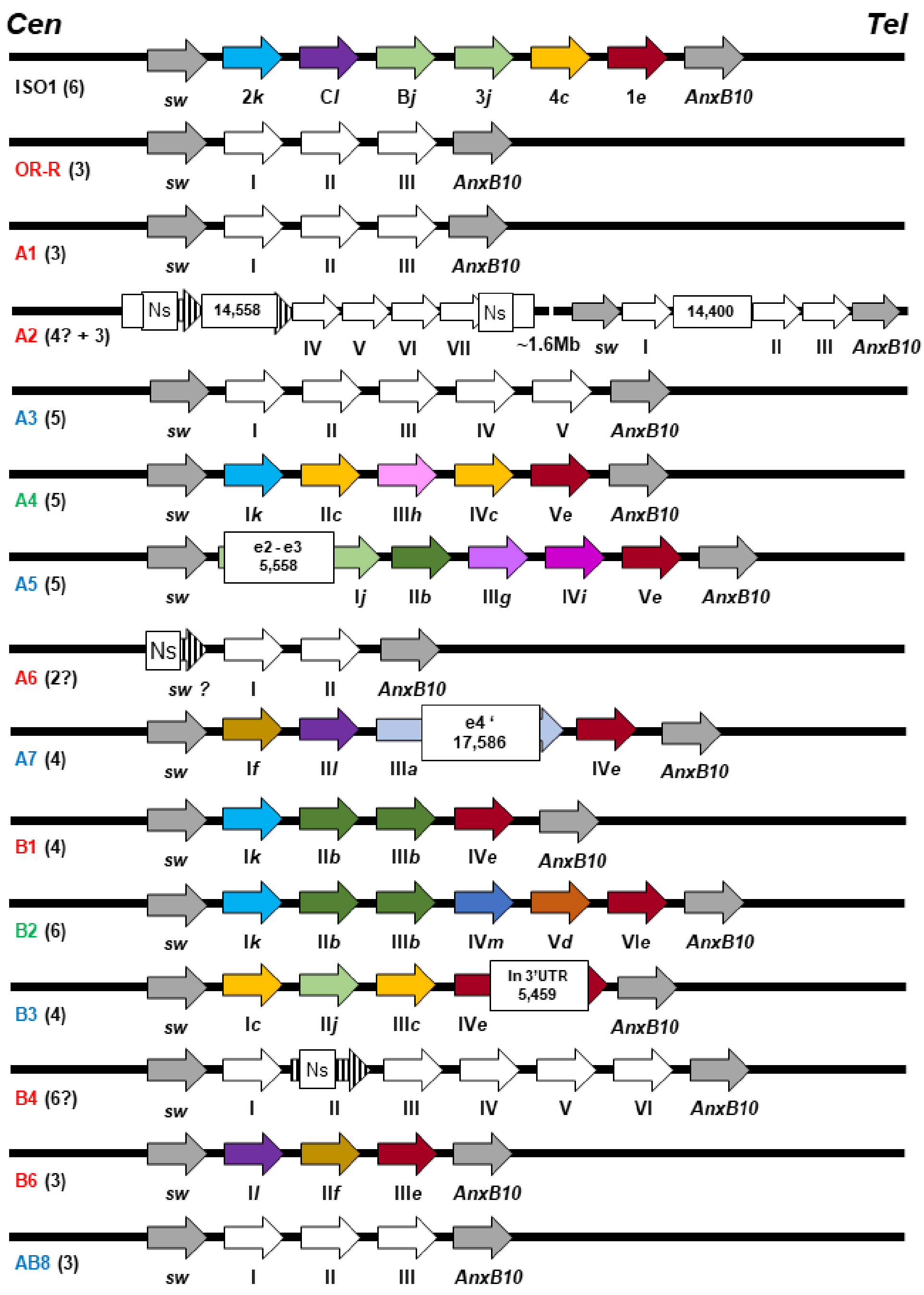

Figure S1. Annotation of the *Sdic* region across 13 populations of the DSPR panel and the wild-type stock OR-R. The most reliable organization of the region at 19C1 on the *X* chromosome in the ISO-1 is provided as a reference (Clifton, et al. 2017). The region is depicted from centromere (Cen) to telomere (Tel), including the flanking genes *sw* and *AnxB10* (grey filled arrows). Population names are color-coded based on the broad continental region where they were collected: green, Africa; red, Americas; and blue, Eurasia. The number of

annotated *Sdic* copies in reference-quality genome assemblies (Chakraborty, et al. 2019; Chakraborty, et al. 2018) is indicated in parentheses next to the name of the population. Arrows filled with vertical lines are partial copies. *Sdic* copies in the ISO-1 strain are named as reported (Clifton, et al. 2017). In the rest of populations, the copy identifiers are roman numerals according to their relative order from *sw* to *AnxB10*. In the most reliable genome assemblies, copies are color coded, and a lower character (*a*-*m*) added to their identifier, both indicating the associated paratype. The size of the TE insertions (solid boxes), as well as their location, are indicated. Ns, assembly gap. e, exon. The apostrophe in the case of A7_III*a* indicates a no longer coding exon, as the STOP codon is upstream of the TE insertion. The *Sdic* region was found unfragmented except for the strains from Bogota (A2) and Georgia (A6). In the case of A2, 6 full *Sdic* copies form two different clusters ~1.6 Mb apart on the *X* chromosome. The distal cluster harbors 3 of the copies, which are flanked by *sw* and *AnxB10.* In contrast, the proximal cluster is flanked by gap assemblies, which in turn are adjacent to TEs. Within this cluster, we found 3 full copies, another copy almost in its entirety, and the remnants of 2 other copies, which are separated by a cluster of TEs. In the case of A6, only two complete *Sdic* copies were found, upstream of which a 1,190 nt long fragment corresponding to the 3' end of either *sw* or an *Sdic* copy is present. This fragment is separated from other genes further upstream of the parental gene *sw,* such as *obst-A*, which is not found in the assembly due to an assembly gap.

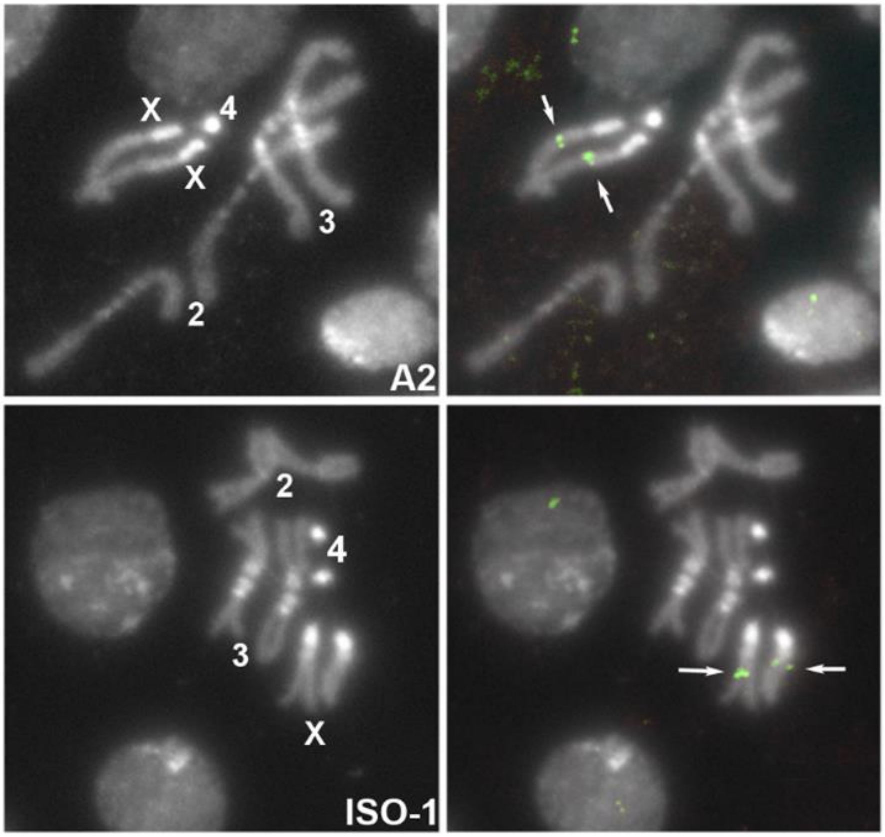

Figure S2. *In situ* hybridization on mitotic chromosomes of A2. A single hybridization signal (arrow) on the *X* chromosome is observed both for A2 (top right) and ISO-1 (bottom right) strains. The squashes shown were obtained from female larvae; squashes from male larvae show the same result discarding any additional copy on the *Y* chromosome.

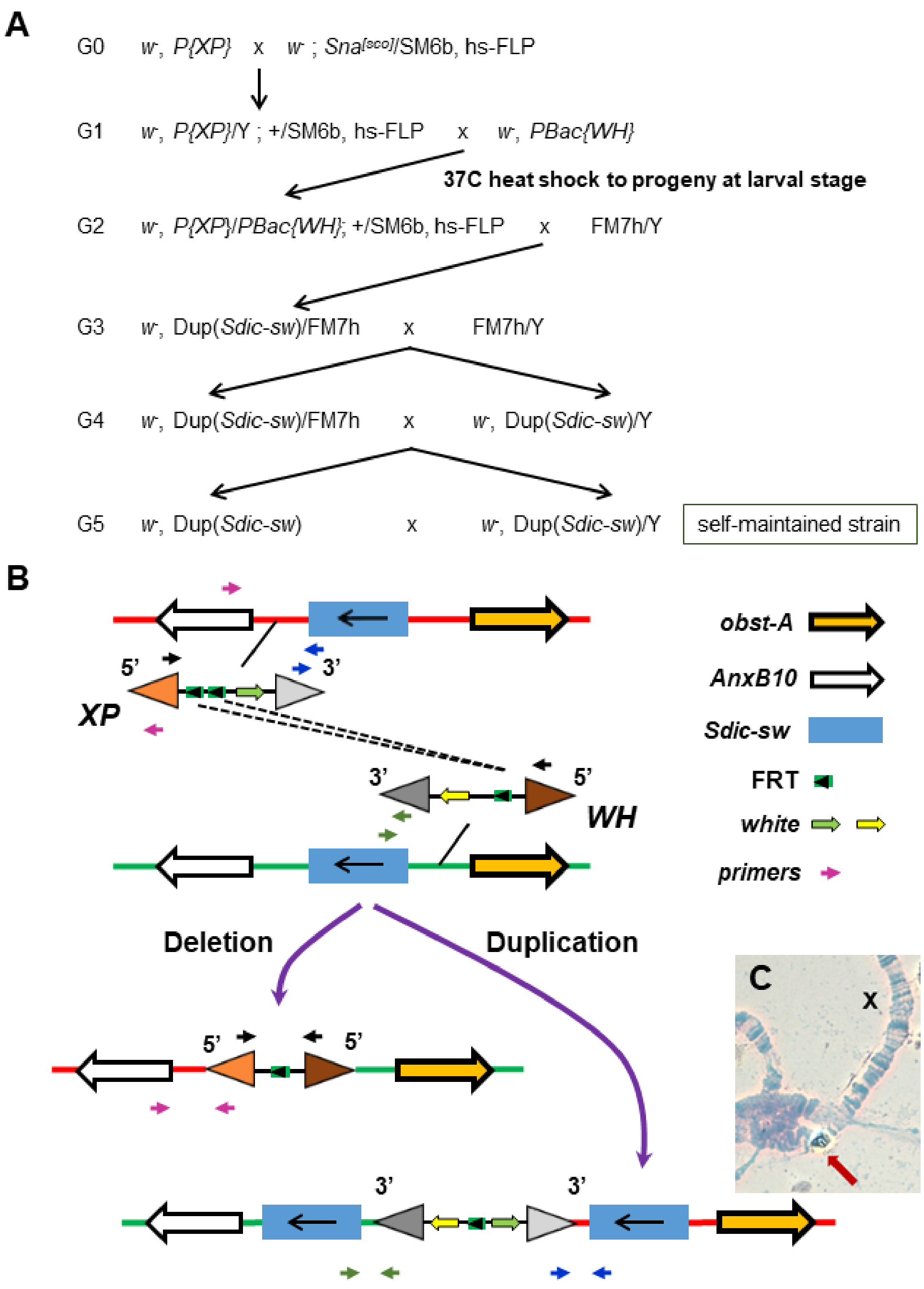

Figure S3. Duplicating the *Sdic* region. (A) Mating scheme followed to duplicate the *Sdic* region through an induced FRT-FLP recombination event. The recombination event took place between the engineered TEs *P{XP}d03903* and *PBac{WH}f02348*, following the same mating scheme used to previously generate the deletion of the *Sdic* region (Yeh, et al. 2012b). The TE *P{XP}d03903* is located in the intergenic region between the genes *AnxB10* and *Sdic1* while *PBac{WH}f02348* is between *sw* and *obst-A.* Therefore, the actual duplication spans from the

Sdic copy adjacent to *AnxB10* to *sw*, inclusive. To discern which females, out of 174 obtained in G3, were actual carriers of the duplication of the *Sdic* region, we used two approaches. First, we visually inspected and PCR-screened the male progeny of 174 females, classifying each female as a duplication or non-duplication bearer based on the eye color of their male progeny. Male progeny was PCR-screened through four controls that provided complementary information (Table S4). Once females carrying the duplication were identified, the mating scheme was continued to make the duplication homozygous. (B) Gene and TE molecular organization along the original TE-bearing chromosomes (top) and those resulting from an ectopic recombination event (bottom). As a duplication event results into a hybrid TE carrying only the 3' ends of the two TEs, two controls (amplicons 1 and 2 respectively in Table S4) were designed to confirm their presence in the PCR screening. Male progeny of these females should give rise to two amplicons (one per 3' end), which were multiplexed in the same PCR reaction. After separating the females presumably carrying the duplication of the *Sdic* region from those carrying its deletion, the females were subjected to two additional PCR controls (amplicons 3 and 4 respectively in Table S4). Amplicon 3 allows to confirm that the *X* chromosome under examination does not carry the deletion or the original chromosome carrying *P{XP}d03903*; the amplicon corresponding to the downstream end of the duplicated *Sdic* region should not be detected. Lastly, a fourth amplicon that corresponds to the hybrid TE that preserves the 5' ends of the two original chromosomes, and should only result from a deletion event, should not be observed either (Yeh, et al. 2012b). The combination from these four PCR controls designated 36 females out of the initial 174 as carriers of a duplicated *Sdic* region. Two of them (2T and 4M) were used in downstream analyses. (C) Chromosomal location of the duplicated *Sdic* region. An extremely intense, single *in situ* hybridization signal can be detected on the *X* chromosome of one of the duplication strains (4M), denoting a local duplication of the *Sdic* region, which is in good agreement with qPCR results.

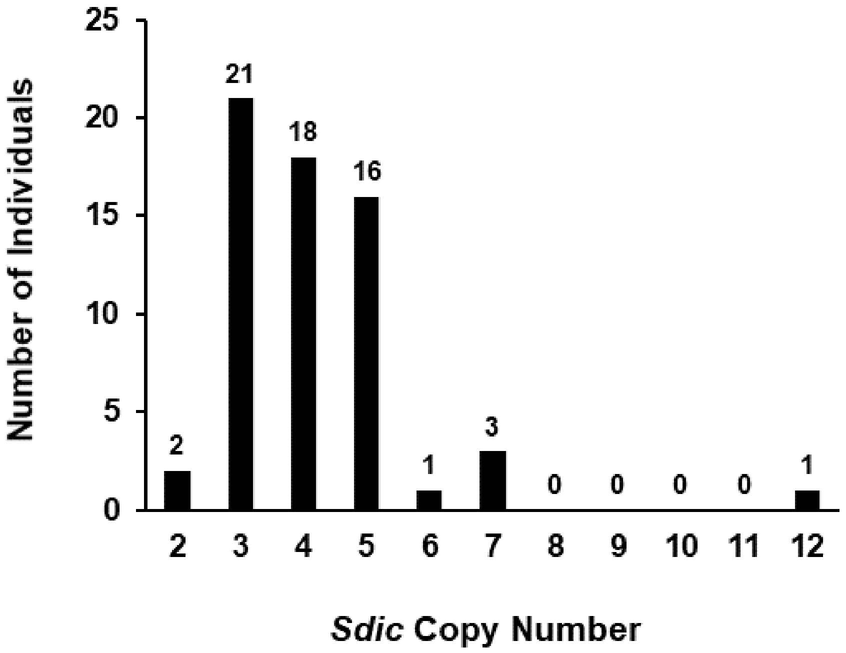

Figure S4. Frequency distribution of *Sdic* CN estimation in haploid embryo genomes from a Zambian population. Each genome dataset corresponds to one female gamete from each strain. Sixty-two haploid embryos were ultimately considered (Material and Methods). The CNVnator program (Abyzov, et al. 2011) was utilized to calculate read-depth average values across a set of synthetic reference genomes derived from A4. The round-off read-depth average values are shown.

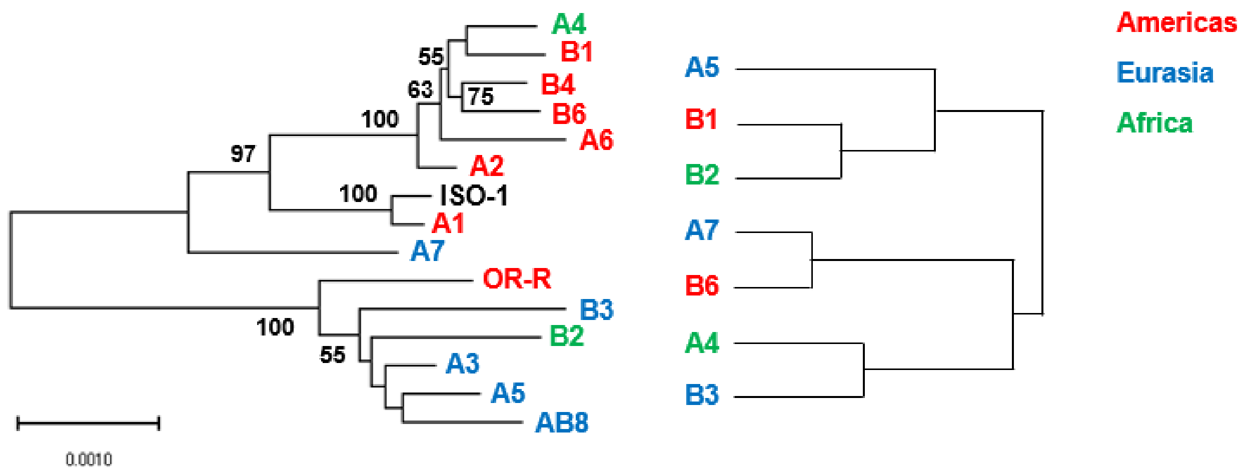

Figure S5. Relationship among strains from the DSPR panel. Left, mtDNA phylogeny of the 14 strains for which their *Sdic* region was annotated in this study plus the reference strain ISO-1. The phylogenetic relationship among strains was inferred by using the Maximum Likelihood method. The tree with the highest log likelihood (-24205.28) is shown. The percentage of replicate trees in which the associated taxa clustered together in the bootstrap test (1,000 replicates) is shown next to the branches when higher than the cut-off value of 50. The tree is drawn to scale, with branch lengths measured in the number of substitutions per site. The continental site of collection for each strain is color-coded as indicated in the legend. The reference strain ISO-1 is shown in black. Right, hierarchical clustering of populations based on their paratype and CN composition. Three principal components that explain ~90% of the total variation were used. The observed patterns of compositional similarity for the *Sdic* region were coincidental with the sorting of the populations based on the Bray-Curtis index (Bray and Curtis 1957), a metric typically used to assess compositional similarity between, for example, two ecological communities based on count data (data not shown). The resulting clustering matches neither the geographical proximity of the collection site of the strains nor, more importantly, the phylogenetic relationship of the populations.

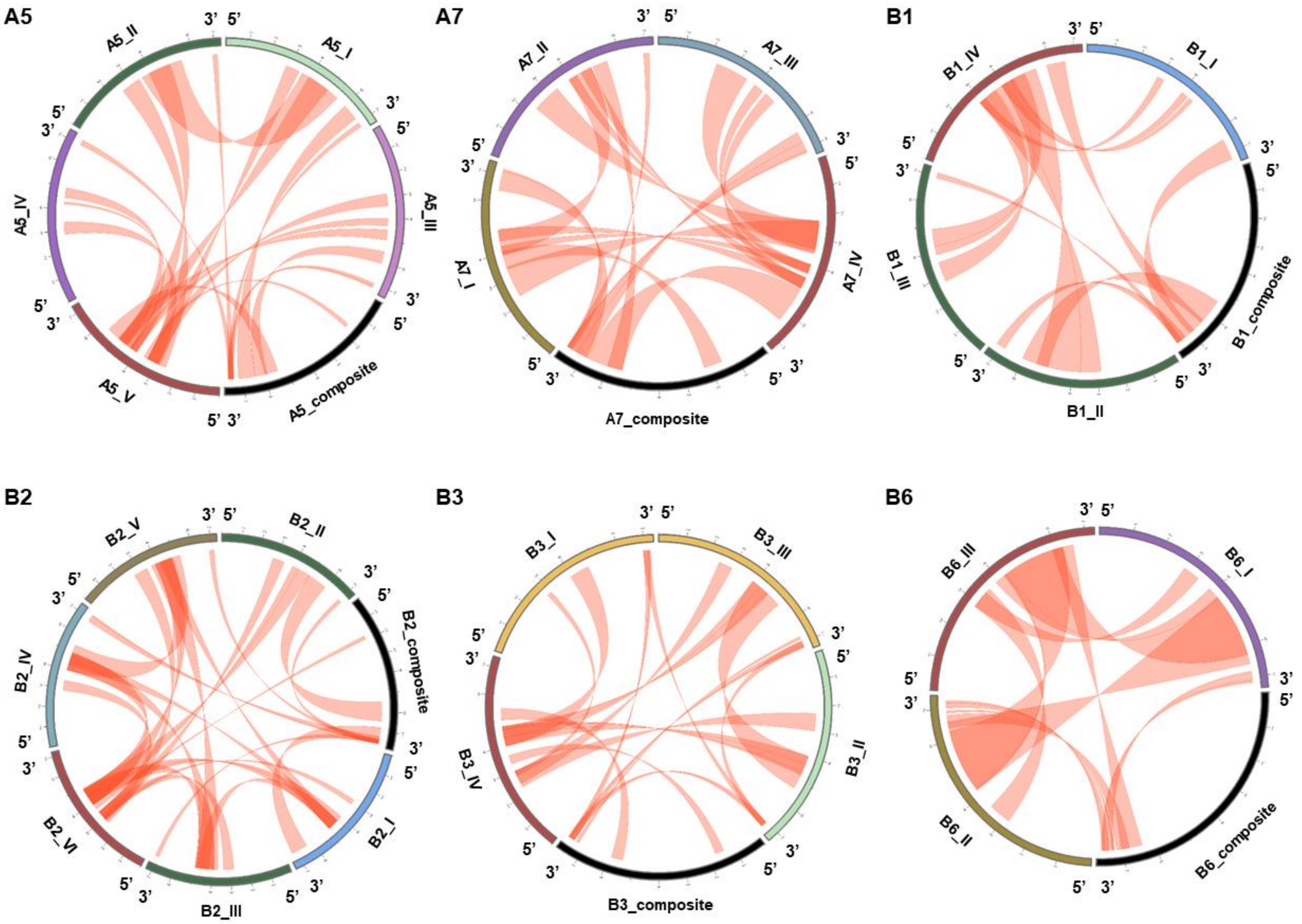

Figure S6. Topology of gene conversion events that have occurred in the *Sdic* region. Circular layouts showing the patterns of gene conversion events occurred between *Sdic* copies and the composite, *i.e.* the fragments from *sw* plus *AnxB10* (black) that align with *Sdic*. The results from GenConv (Sawyer 1989) are graphed for the six strains not shown in Fig. 4C.

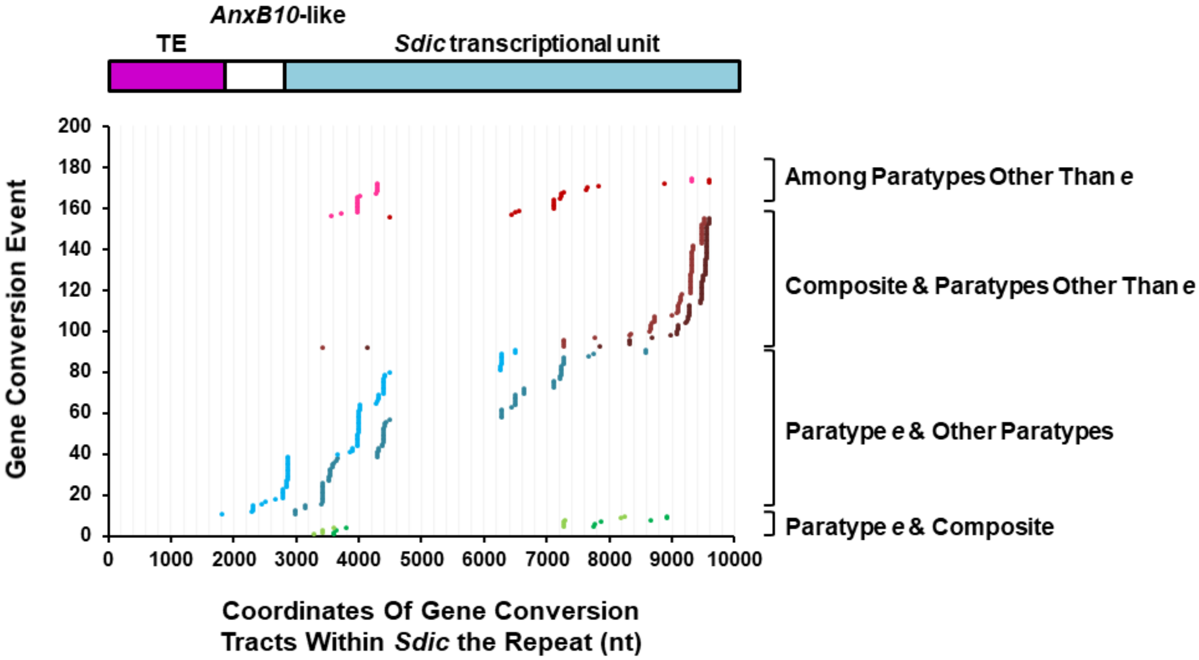

Figure S7. Topological occurrence of gene conversion events along *Sdic* repeats. Plot of coordinates of 174 gene conversion tracts involving different *Sdic* paratypes and the parental gene *sw* as detected with GeneConv across strains. Coordinates for different types of events are color-coded (see legend on the right). Start and end coordinates, in lighter and darker tones respectively, of the same gene conversion event project onto the same value of the *y*-axis. All tracts detected across strains are shown. Outer events (or fragments according to the nomenclature of GeneConv) are not shown.

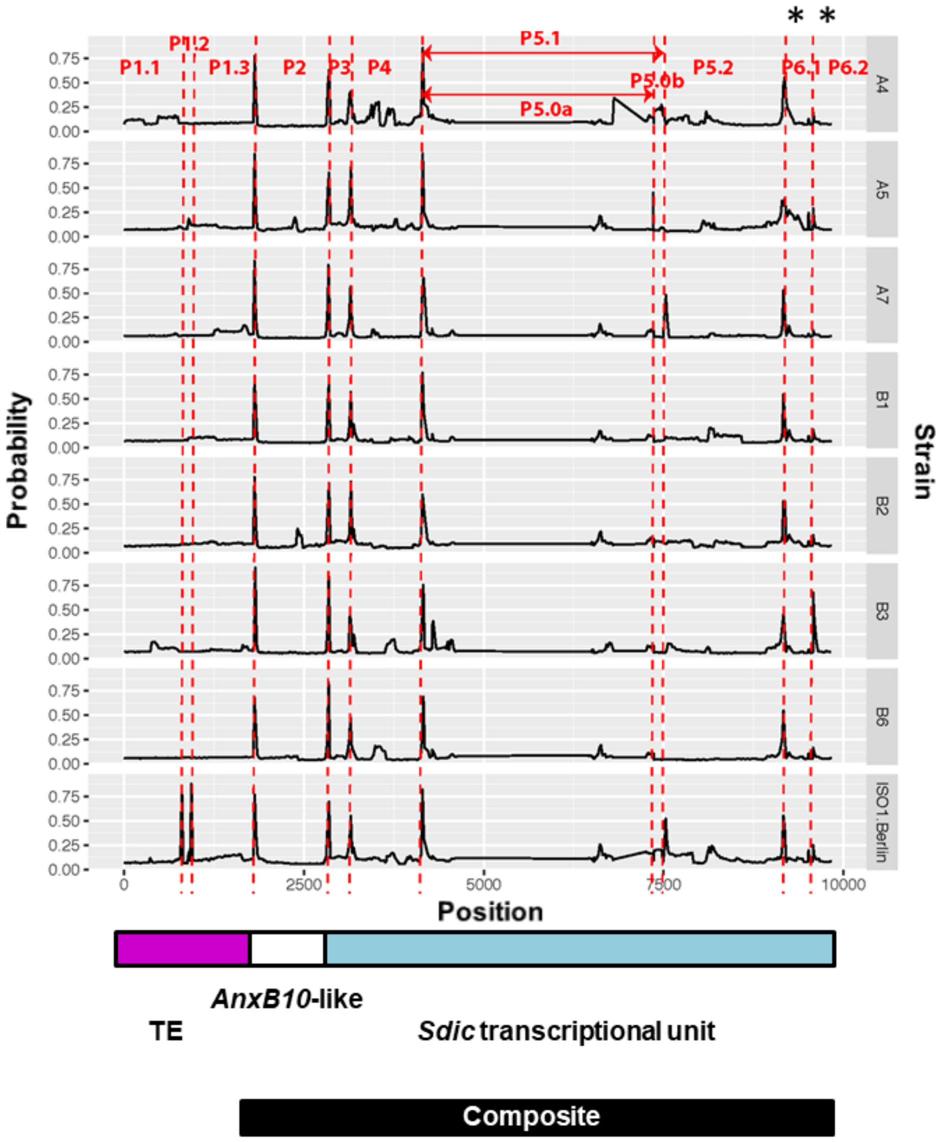

Figure S8. Breakpoint distribution along the *Sdic* repeat across the strains. Breakpoint location inferred with AGC (O'Fallon 2013). The location of highly supported breakpoints is indicated with a dotted red line and the resulting partitions numbered accordingly from 5' to 3' (P1-P6). The partitions for which there is strong evidence of the action of positive selection are indicated with asterisks (top). Distance in nucleotides relative to the 5' end of the *Sdic* repeat can be interpolated from the *x*-axis. The composite, *i.e.* the fragments from *sw* plus *AnxB10* (black) that align with *Sdic,* is shown at the bottom. *y*-axis, probability of breakpoint occurrence.

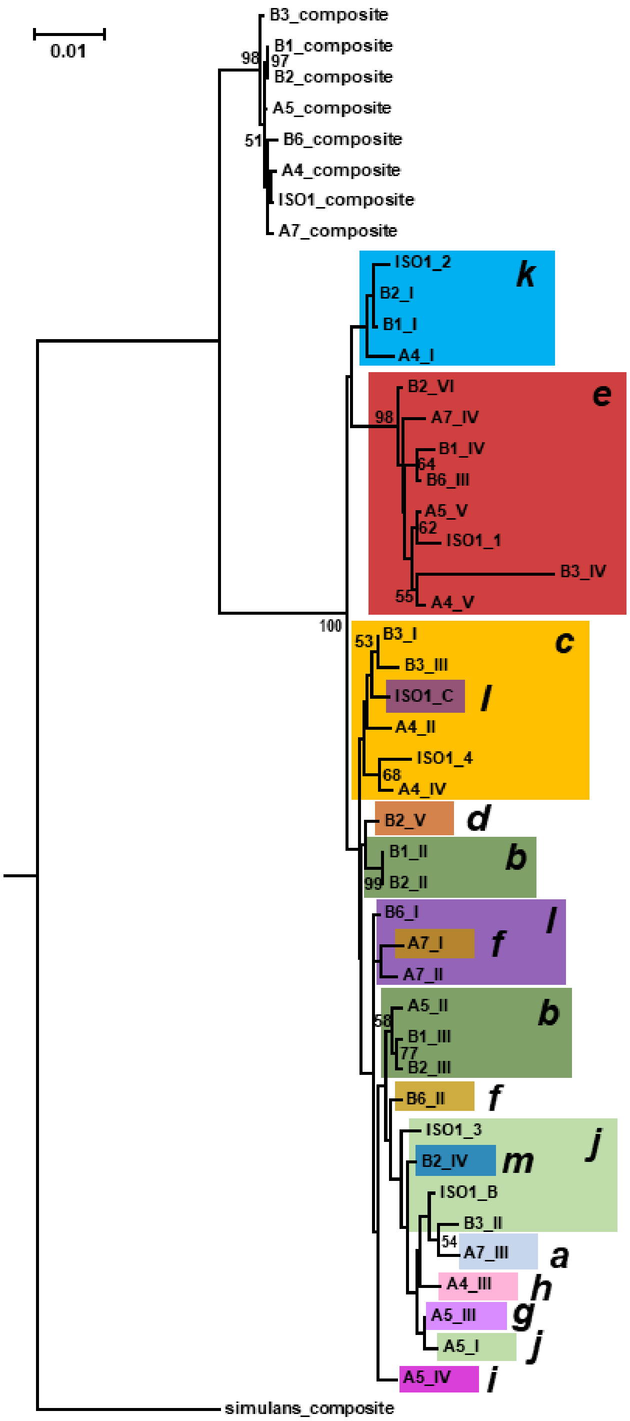

Figure S9. Phylogenetic relationships among *Sdic* copies. The copies considered are the 31 from the strains A4, A5, A7, B1, B2, B3, and B6, plus the six copies from the reference strain ISO-1. Copy nomenclature is as in Fig. 1; also copies that belong to the same paratype are shaded according to the color code in that same figure. The phylogeny shown was inferred with RAxML 8.1.2 under a GTRGamma model of sequence evolution. The composites, *i.e.* the

constructs generated with the alignable stretches of DNA sequence between *Sdic* and the parental genes *sw* and *AnxB10*, from each strain were also included in the analysis. The equivalent composite was generated for *D. simulans* according to the available information in FlyBase (Hu, et al. 2013). The percentage of replicate trees in which the associated copies clustered together in the bootstrap test (1,000 replicates) is shown next to the branches when higher than the cut-off value of 50. Copies representing paratype *e,* the ones for which is found the strongest support for the most recent action of positive selection, form a very distinctive clade. The same conclusion and a very similar overall tree topology are found when inferring the phylogeny of the copies under a best-fit substitution model.

Table S1. Strains used in empirical work

Strain	Stock Number	Genotype	Comment	Source	Reference
w^{1118}	DSK001	w^{1118} ; 2_{iso}; 3_{iso}	Isogenic laboratory background for *P* insertions	DrosDel Collection	(Ryder, et al. 2004)
P{XP}d03903	d03903	w^{1118} , *P{XP}d03903*; 2_{iso}; 3_{iso}	P element donor	Exelixis Collection	(Parks, et al. 2004)
PBac{RB}f02348	f02348	w^{1118} , *PBac{RB}f02348*; 2_{iso}; 3_{iso}	P element donor	Exelixis Collection	(Parks et al. 2004)
SM6b, 70FLP ry^{+}	123-58	$w/y^{+}Y$; sna^{Sco}/SM6b, P{70FLP, $ry^{+t7.2}$}7	Flippase source	Cambridge Fly Facility	na
FM7h/CB-6411-3	123-65	*FM7d, w oc ptg/P{RS3}l(1)CB-6411-3*	Balancer	Cambridge Fly Facility	na
A-	na	w^{1118}, Df(1)FDD-0053249^{A}/FM7h)	Deficiency	Own stock	(Clifton, et al. 2017)
E^{-}	na	w^{1118}, Df(1)FDD-0053249^{E}/FM7h)	Deficiency	Own stock	(Clifton et al. 2017)
2T	na	w^{1118}, Dp(1;1)Sdic:sw^{2T}	Duplication	Own stock	This work
4M	na	w^{1118}, Dp(1;1)Sdic:sw^{4M}	Duplication	Own stock	This work
ISO-1	2057	y^{1}; cn^{1}; bw^{1}; sp^{1}	Reference	BDSC	(Adams, et al. 2000)
OR-R	W-20	+; +; +	Wildtype	Cambridge Fly Facility	na
A1	1	+; +; +	Wildtype	BDSC	(King, et al. 2012a)
A2	3841	+; +; +	Wildtype	BDSC	(King et al. 2012a)
A3	3844	+; +; +	Wildtype	BDSC	(King et al. 2012a)
A4	3852	+; +; +	Wildtype	BDSC	(King et al. 2012a)
A5	3875	+; +; +	Wildtype	BDSC	(King et al. 2012a)
A6	3886	+; +; +	Wildtype	BDSC	(King et al. 2012a)
A7	14021-0231.7	+; +; +	Wildtype	DSSC	(King et al. 2012a)
B1	3839	+; +; +	Wildtype	BDSC	(King et al. 2012a)
B2	3846	+; +; +	Wildtype	BDSC	(King et al. 2012a)
B3	3864	+; +; +	Wildtype	BDSC	(King et al. 2012a)
B4	3870	+; +; +	Wildtype	BDSC	(King et al. 2012a)
B6	14021-0231.1	+; +; +	Wildtype	DSSC	(King et al. 2012a)

BDSC, Bloomington *Drosophila* Stock Center; DSSC (*Drosophila* Species Stock Center), UC San Diego.

Table S2. *Sdic* copy number estimates across 22 genotypes and three methodologies

Strain or Progeny	Collection Site §	Annotation (Region Size *)	CNVnator †	qPCR ‡
ISO-1	na	6 (57.2 kb) *	6	6 (M) / 12 (F)
w^{1118}	na	na	na	6
A-	na	na	na	0
E-	na	na	na	0
2T	na	na	na	12
4M	na	na	na	12
I: 4M (F) x w^{1118} (M)	na	na	na	12 (M)
II: 4M (F) x w^{1118} (M)	na	na	na	18 (F)
III: w^{1118} (F) x 4M (M)	na	na	na	6 (M)
IV: w^{1118} (F) x 4M (M)	na	na	na	18 (F)
OR-R	Roseburg, Oregon	3 (34.1 kb)	6	6
A1	Canton, Ohio	3 (33.8 kb)	6	6
A2	Bogota, Colombia	7 (48.9 kb)	5	5
A3	Barcelona, Spain	5 (49.0 kb)	6	6
A4	Kariba Dam, South Africa	5 (49.5 kb)	5	5
A5	Athens, Greece	5 (56.7 kb)	5	5
A6	Red Top Mountain, Georgia	2 (18.0 kb)	4	4
A7	Ken-Ting, Taiwan	4 (59.8 kb)	4	4
B1	Bermuda	4 (41.7 kb)	4	4
B2	Cape Town, South Africa	6 (57.0 kb)	6	6
B3	Israel	4 (48.7 kb)	4	4
B4	Riverside, California	6 (58.3 kb)	5	5
B6	Ica, Peru	3 (33.9 kb)	3	3
AB8	Samarkand, Uzbekistan	3 (34.1 kb)	5	na

§ For natural populations only. With the exception of OR-R, the rest correspond to the founder strains of the Drosophila Synthetic Population Resource or DSPR (King, et al. 2012a).

* From the first nucleotide at the 5'UTR of *sw* to the last nucleotide at the 3'UTR of *AnxB10*.

† Rounded-off average read-depth values.

‡ Rounded-off qPCR estimates derived from the difference between the amplicons sw-*Sdic* and *sw* only. In males unless specified (M, males; F, females).

Table S3. Normalized read-depth values obtained with CNVnator for the strains of the DSPR panel

	Reference Genome *											Rounded Off	CN
**Strain**	**ISO1_1**	**ISO1_2**	**ISO1_3**	**ISO1_4**	**ISO1_5**	**ISO1_6**	**A4_1**	**A4_2**	**A4_3**	**A4_4**	**A4_5**	**Average**	**Estimate †**
ISO1	7.401	3.173	7.447	7.429	7.523	7.385	7.543	6.675	7.613	7.555	7.527	7	6
ORR	7.678	7.831	7.784	7.725	2.359	2.302	7.485	7.498	7.505	7.490	7.513	7	6
A1	6.794	6.879	6.864	6.801	6.805	6.582	6.476	6.504	6.470	6.467	6.143	7	6
A2	6.053	6.115	6.098	6.047	6.121	4.748	5.905	5.930	5.930	5.894	5.743	6	5
A3	6.769	6.798	6.724	6.705	6.797	5.224	6.707	6.703	6.705	6.706	6.345	7	6
A4	6.430	6.533	5.639	6.461	5.665	6.243	6.207	6.248	6.259	6.200	5.984	6	5
A5	6.064	6.177	6.141	6.110	6.175	5.927	5.859	5.893	5.868	5.851	5.640	6	5
A6	3.744	6.361	5.575	6.243	6.317	5.616	6.108	3.779	6.062	3.747	5.872	5	4
A7	5.249	5.303	5.288	5.251	5.296	5.145	5.145	4.756	4.828	4.703	4.405	5	4
B1	4.833	4.890	4.867	4.845	4.887	4.664	4.459	4.611	4.589	4.563	4.271	5	4
B2	7.069	7.253	7.224	7.168	7.259	6.821	6.903	7.004	6.941	6.948	6.630	7	6
B3	5.020	5.103	5.084	5.049	5.104	4.468	4.851	4.876	4.861	4.838	4.670	5	4
B4	5.983	6.044	5.934	5.989	6.047	5.487	5.852	5.879	5.674	5.847	5.592	6	5
B6	4.339	4.427	4.395	4.379	4.416	4.333	4.344	4.382	4.372	4.331	4.273	4	3
AB8	6.073	6.144	6.133	6.097	6.163	5.941	5.772	5.803	5.874	5.765	5.734	6	5

DSPR, Drosophila Synthetic Population Resource.

* Each reference genome used carries one single *Sdic* copy (either from the ISO-1 or A4 strain) and lacks the two parental flanking genes *sw* and *AnxB10.* The number in the ID of the reference genome denotes the order of the *Sdic* copy in the original strain between the flanking genes, specifically from *sw* to *AnxB10*. For example, A4_5 refers to the fifth copy starting from *sw*, *i.e.* the copy adjacent to *AnxB10* in the A4 strain.

† The CN estimate is calculated as the rounded off average minus one due to the contribution of reads from *sw* and *AnxB10* to the read-depth estimates obtained with CNVnator.

Table S4. Primers used

Amplicon # (Description)	Forward Primer (5'-3')	Reverse Primer (5'-3')	Ta (C)	Size (nt)	Experiment
1 (Unaltered *WH*3' end)	sw-WH_*F* TGTTTGATTAAAATGCTGAGTGTG	WH3'+ CCTCGATATACAGACCGATAAAAC †	59	621	Dup.
2 (Unaltered *XP*3' end)	XP3'- TACTATTCCTTTCACTCGCACTTATTG †	XP-Sdic1_*R* TAGAACTACCCGCATATTTGATTG	59	300	Dup.
3 (Unaltered distal junction)	AnxB10-intron1_*F* TCTCTAGCCTGGCAATCCAATC	XP5'-*R* AGCCTTCCACTGCGAATCATT §	58	900	Dup.
4 (Hybrid TE deletion)	XP5'+ AATGATTCGCAGTGGAAGGCT *	WH5'- GACGCATGATTATCTTTTACGTGAC *	55	1,400	Dup.
5 (*Sdic/sw*)	AACGGATTCACCTCCAAGC	GATCTCGAGTGGTGTGATGG	60	93	qPCR
6 (*sw*)	GCGAGAAGGAGATCAAGGAC	CTGATCCTTGTCGATGCCTG	60	74	qPCR
7 (*TPI*)	AGGCAACTGGAAGATGAACG	GATGACCACCTCCGTGTTG	60	97	qPCR
8 (*Gapdh2*)	CAAGCAAGCCGATAGATAAAC *	GTCAAATCGACCACGGAAA *	52	762	qRT-PCR
9 (*Sdic*)	CGTATTCTACTTTGAGCGGCG	GGAATGTTCGTAGCCTGCAC	60	76	qRT-PCR
10 (*clot*)	GAGCGGGCATACTGGAAG	GCAACAGAGTGGGCAAGAAG	60	82	qRT-PCR
11 (*Sdic/sw*)	TGCAGTTTCCCCTGATTTCTT *	AGACGAAGAAGAACGCGTAATG *	54.3	2,253	In situ
12 (*Sdic/sw*)	CATTTGATGCCCAAGGAGAC	AGGAAGAGGTGGCCAAAGTC	60	1,434	FISH
13 (TE_A2_uj)	CAAGATGAACCAGAGCGATG	GCACTTGGCTGTCACAAGAG	60	684	PCR
14 (TE_A2_dj)	CACAAGCGGTTTCCTTTAGC	TTGGGCTCTTTCAGTTGAGG	60	716	PCR
15 (TE_A7_uj)	TCAATCCCAACCTGATCCTC	CACAAGCGGTTTCCTTTAGC	60	886	PCR
16 (TE_A7_dj)	CGCGTCAGCATTGTTCATAC	ACCTCCGTGTCTTGGTTGAG	60	610	PCR
17 (TE_A5_uj)	CAATCTGTCCATCCACATGC	ATTGCATTTGGCTAGCTTGG	60	404	PCR
18 (TE_A5_dj)	AGTCCAAGCTAGCCAAATGC	GGAGAGAAGGAGCATTGCAG	60	623	PCR
19 (TE_B3_uj)	AGCCGCTGTACTCCTTTGAG	CTGCCCTCTTTCAACGCTAC	60	748	PCR
20 (TE_B3_dj)	TGACTAAGGACAACGCCAAG	GCTTTATGCCGAAAGAGTCG	60	659	PCR

Dup., engineered duplication experiment. In situ, *in situ* hybridization on polytene chromosomes. FISH, *in situ* hybridization on mitotic chromosomes. Ta, annealing temperature. uj, upstream junction; dj, downstream junction. Unless indicated, primer design was performed in this study.

† (Parks, et al. 2004).

§ Reverse complementary of the XP5'- primer when combined with the primer WH5'+ (Parks, et al. 2004).

* (Yeh, et al. 2012b).

Table S5. Pearson's correlation coefficient among CN estimates obtained with different methodologies

	Genome Annotation	qPCR	CNVnator
**Genome Annotation**	-	0.3369 (*P* = 0.2603)	0.3141 (*P* = 0.2741) *
**qPCR**		-	1 (*P* < 0.0001) * ‡
**CNVnator**			-

* In comparisons involving qPCR values, AB8 was omitted.

‡ Pearson's correlation coefficient prior to rounding-off CNVnator and qPCR original values was 0.9720.

Table S6. Logistic regression analysis to evaluate the relevance of different assembly metrics in the faithful recapitulation of the *Sdic* region

	Variable	Deviance	AIC	LRT	*P*
Genome-wide analysis					
(AIC = 21.41; r2ML = 0.1067)	Total_seqs	17.825	21.825	1.584	0.208
	Coverage	17.828	21.828	1.580	0.209
	NR50	17.837	21.837	1.572	0.210
	Assembly_N50	18.523	22.523	0.886	0.347
	canu_N50	18.881	22.881	0.527	0.468
	DBG2OLC_N50	19.333	23.333	0.076	0.783
Local analysis					
(AIC = 17.07; r2ML = 0.3996)	Coverage	20.728	22.728	7.653	0.006
	Size_uncorrected	11.915	17.915	1.160	0.281
	Size_corrected	12.345	18.345	0.730	0.393
	Assembly_N50	12.605	18.605	0.470	0.493
	NR50	12.669	18.669	0.406	0.524

AIC, Akaike information criterion; r2ML, maximum likelihood pseudo r^2; LRT, likelihood ratio test.
Model choice by AIC was done using the ISLR R package by applying a forward stepwise algorithm. For the genome-wide analysis, the values of the different variables across assemblies were taken from (Chakraborty, et al. 2019). For the local analysis, the values used are those in Table S7. Size corrected or uncorrected refer to the size of the region as interpolated from CNVnator estimates.

N50 refers to the length of the smallest contig, after ranking them from longest to smallest, such that the sum of the contig lengths up to it spans 50% of the total assembly size. NR50s refers to the median read length above which half of the total coverage is contained.

Table S7. Analysis of sequencing reads associated with the *Sdic* region across datasets

Strain	Sequencing Dataset	Sequencing Reads Including Particular Gene Entities * Only *Sdic*	*Sdic* and *sw*	*Sdic* and *AnxB10*	*Sdic*, *sw*, *AnxB10*	Total	NR50 (kb)	Local Coverage (x)
ISO1	Nanopore_Corrected	75	16	19	0	110	11.84	19.6
ISO1	Nanopore_Uncorrected	84	14	14	0	112	12.184	18.9
ISO1	SMRT	104	16	17	0	137	13.994	33.2
ORR	SMRT	106	11	11	0	128	12.069	26.8
A1	SMRT	135	5	18	0	158	12.1	30.1
A2	SMRT	98	6	18	0	122	13.576	22.5
A3	SMRT	60	8	14	0	82	15.339	20.6
A4	SMRT	178	41	56	0	275	17.885	93.1
A5	SMRT	133	16	21	0	170	13.228	42.3
A6	SMRT	39	5	9	0	53	14.984	17.6
A7	SMRT	172	20	31	0	223	14.838	57.5
B1	SMRT	52	9	14	0	75	17.924	33.0
B2	SMRT	103	6	29	0	138	14.895	37.6
B3	SMRT	67	9	16	0	92	15.717	29.2
B4	SMRT	108	5	9	0	122	12.242	31.8
B6	SMRT	74	13	23	0	110	12.257	39.1
AB8	SMRT	90	15	20	0	125	17.283	41.0

* Either partially or entirely. Determined by BLASTn using diagnostic regions of the genes of interest.

Table S8. CNVnator results for strains from six different populations

		Illumina	Reference Genome †						Rounded-Off	CN
Population *	Strain	Library ID	A4_1	A4_2	A4_3	A4_4	A4_5	Average	Average	Estimate ‡
ZW	ZH23	SRX765992	6.547	6.562	6.579	6.572	6.484	6.549	7	6
ZW	ZH26	SRX766078	8.583	8.617	8.622	8.585	8.412	8.564	9	8
ZW	ZH33	SRX766073	8.437	8.473	8.516	8.500	8.456	8.476	8	7
ZW	ZH42	SRX766069	7.184	7.289	7.328	7.303	7.168	7.254	7	6
ZW	ZS10	SRX765993	10.614	10.616	10.628	10.633	10.597	10.618	11	10
ZW	ZW09	SRX766079	7.864	8.020	8.023	8.000	7.779	7.937	8	7
ZW	ZW139	SRX766075	7.380	7.404	7.416	7.388	7.424	7.402	7	6
ZW	ZW140	SRX766074	7.837	7.842		7.846	7.677	7.800	8	7
ZW	ZW142	SRX766072	8.082	8.120	8.189	8.201	8.017	8.122	8	7
ZW	ZW155	SRX766071	9.282	9.295	9.345	9.301	9.188	9.282	9	8
ZW	ZW177	SRX765990	6.049	6.058	6.085	6.058	6.042	6.058	6	5
ZW	ZW185	SRX766096	8.944	9.062	9.114	9.096	9.033	9.050	9	8
T	T05	SRX766109	6.448	6.494	6.497	6.475	6.449	6.473	6	5
T	T07	SRX766106	6.436	6.462	6.447	6.447	6.390	6.436	6	5
T	T09	SRX766105	7.929	8.042	8.027	8.022	7.882	7.980	8	7
T	T10	SRX766104	7.669	7.778	7.778	7.766	7.677	7.734	8	7
T	T14A	SRX766102	7.416	7.409	7.557	7.527	7.421	7.466	7	6
T	T22A	SRX766114	6.816	6.846	6.866	6.820	6.721	6.814	7	6
T	T23	SRX766101	7.421	7.448	7.495	7.656	7.334	7.471	7	6
T	T24	SRX766115	6.613	6.703	6.701	6.665	6.453	6.627	7	6
T	T25A	SRX766112	7.276	7.385	7.374	7.291	7.282	7.322	7	6
T	T29A	SRX766107	7.750	7.774	7.768	7.770	7.733	7.759	8	7
T	T30	SRX766111	6.937	6.965	7.002	6.853	6.837	6.919	7	6
T	T35	SRX766127	7.505	7.551	7.534	7.544	7.486	7.524	8	7
T	T36B	SRX766122	6.633	6.632	6.674	6.635	6.580	6.631	7	6
T	T39	SRX766137	7.559	7.570	7.611	7.574	7.471	7.557	8	7
T	T43A	SRX766134	4.682	4.695	4.710		4.652	4.685	5	4
T	T45B	SRX766129	7.598	7.637	7.641	7.614	7.593	7.617	8	7
N	N01	SRX766128	5.851	5.865	5.871	5.858	5.844	5.858	6	5
N	N02	SRX766120	5.755	5.774	5.848	5.799		5.794	6	5
N	N03	SRX766118	6.334	6.496	6.478	6.493		6.450	6	5
N	N04	SRX766117	7.445	7.610	7.628	7.617		7.575	8	7
N	N07	SRX766132	5.336	5.372	5.322	5.244	5.253	5.305	5	4
N	N10	SRX766131	8.437	8.611	8.663	8.620	8.483	8.563	9	8

Table S8. CNVnator results for strains from six different populations

		Illumina	Reference Genome †						Rounded-Off	CN
Population *	Strain	Library ID	A4_1	A4_2	A4_3	A4_4	A4_5	Average	Average	Estimate ‡
N	N11	SRX766126	8.149	8.156	8.309	8.273	8.174	8.212	8	7
N	N13	SRX766133	7.798	7.828	7.795	7.838	7.580	7.768	8	7
N	N14	SRX766130	6.510	6.544	6.508	6.514	6.446	6.505	7	6
N	N15	SRX766125	5.535	5.454	5.571	5.427		5.497	5	4
N	N16	SRX766124	5.804	5.894	5.875	5.876	5.834	5.857	6	5
N	N17	SRX766121	5.757	5.826	5.827	5.850	5.807	5.813	6	5
N	N18	SRX766136	7.600	7.603	7.482	7.465	7.555	7.541	8	7
N	N19	SRX766123	6.793	6.884	6.861	6.745		6.821	7	6
N	N22	SRX766191	6.889	6.914	6.906	6.890		6.900	7	6
N	N23	SRX766178	7.671	7.776	7.776	7.751		7.744	8	7
N	N25	SRX766177	6.233	6.233	6.266	6.201	6.151	6.217	6	5
N	N29	SRX766189	9.283	9.316	9.370	9.311	9.040	9.264	9	8
N	N30	SRX766187	6.472	6.494	6.472	6.479		6.479	6	5
I	I03	SRX766185	5.698	5.723	5.746	5.743	5.706	5.723	6	5
I	I07	SRX766184	7.811	7.836	7.852	7.828	7.836	7.833	8	7
I	I13	SRX766183	6.796	6.831	6.743	6.731	6.693	6.759	7	6
I	I17	SRX766182	7.321	7.430	7.406	7.403		7.390	7	6
I	I22	SRX766181	7.380	7.486	7.470	7.462	7.383	7.436	7	6
I	I23	SRX766192	6.893	6.934	6.937	6.904		6.917	7	6
I	I24	SRX766190	8.957	8.997	9.029	8.973	8.947	8.981	9	8
I	I29	SRX766188	6.641		6.671	6.657	6.622	6.648	7	6
I	I33	SRX766186	9.616	9.623	9.612	9.667	9.631	9.630	10	9
I	I34	SRX766180	9.167	9.193	9.239	9.195	9.170	9.193	9	8
I	I35	SRX766179	7.737	7.752	7.799	7.782	7.710	7.756	8	7
I	I38	SRX766204	8.942	8.952	8.929	8.963	8.881	8.933	9	8
B	B04	SRX766199	6.949	6.965	6.968	6.993	6.955	6.966	7	6
B	B10	SRX766196	5.271	5.306	5.334	5.287	5.285	5.297	5	4
B	B11	SRX766193	6.227	6.288	6.320	6.294	6.287	6.283	6	5
B	B12	SRX766201	6.073	6.099	6.128	6.105	6.033	6.088	6	5
B	B23	SRX766200	7.166	7.117	7.182	7.104	7.155	7.145	7	6
B	B28	SRX766198	6.907	6.965	6.992	6.962	6.853	6.936	7	6
B	B38	SRX766195	6.762	6.767	6.812	6.800	6.826	6.793	7	6
B	B42	SRX766203	5.669	5.691	5.696	5.695	5.684	5.687	6	5
B	B43	SRX766206	6.620	6.638	6.656	6.641	6.580	6.627	7	6

Table S8. CNVnator results for strains from six different populations

Population *	Strain	Illumina Library ID	Reference Genome † A4_1	A4_2	A4_3	A4_4	A4_5	Average	Rounded-Off Average	CN Estimate ‡
B	B54	SRX766194	6.253	6.259	6.266	6.261	6.244	6.257	6	5
B	B59	SRX766197	6.646	6.716	6.719	6.664		6.686	7	6
ZM	ZI10_1-HE	SRR203502	8.068	8.138		8.167	8.319	8.173	8	7
ZM	ZI152_1-HE	SRR326790	4.160	4.018	4.057	4.157	4.223	4.123	4	3
ZM	ZI173_1-HE	SRR203330	5.170	5.185	5.227	5.216	5.279	5.215	5	4
ZM	ZI177_1-HE	SRR326796	4.015	4.057	4.024	4.114	4.140	4.070	4	3
ZM	ZI181_1-HE	SRR203069	4.475	4.444		4.506	4.550	4.494	4	3
ZM	ZI184_1-HE	SRR203068	4.176	4.166	4.235	4.231	4.296	4.221	4	3
ZM	ZI188_1-HE	SRR202123	5.918	5.918	5.948	5.959	6.044	5.957	6	5
ZM	ZI194_1-HE	SRR203319	5.861	5.929	5.938	5.967	6.021	5.943	6	5
ZM	ZI196_1-HE	SRR203467	5.681		5.845	5.725	5.834	5.771	6	5
ZM	ZI197N_1-HE	SRR342395	4.605	4.566	4.623	4.628	4.684	4.621	5	4
ZM	ZI199_1-HE	SRR203468	6.066	6.092	6.135	6.172	6.094	6.112	6	5
ZM	ZI207_1-HE	SRR202075	4.321	4.298	4.404	4.544		4.392	4	3
ZM	ZI212_1-HE	SRR204012	5.991	5.993	6.045	6.085	6.059	6.035	6	5
ZM	ZI216N_1-HE	SRR203328	4.927	4.945	4.971	4.977	4.945	4.953	5	4
ZM	ZI226_1-HE	SRR203348	5.145	5.130	5.197	5.222	5.285	5.196	5	4
ZM	ZI227_1-HE	SRR202126	5.522	5.552	5.594	5.647	5.670	5.597	6	5
ZM	ZI228_1-HE	SRR203064	3.310	3.364	3.354	3.400	3.436	3.373	3	2
ZM	ZI232_1-HE	SRR202076	3.588	3.584	3.626	3.647	3.643	3.618	4	3
ZM	ZI241_1-HE	SRR326798	5.108	5.126	5.151	5.179	5.285	5.170	5	4
ZM	ZI252_1-HE	SRR203349	6.676	6.762	6.759	6.831	6.798	6.765	7	6
ZM	ZI253_1-HE	SRR203350	6.120	6.076		6.207	6.234	6.159	6	5
ZM	ZI281_1-HE	SRR342393	5.529	5.524	5.564	5.567	5.603	5.558	6	5
ZM	ZI284_1-HE	SRR654554	5.423	5.501	5.513	5.540	5.568	5.509	6	5
ZM	ZI295_1-HE	SRR202099	6.681	6.675		6.664	6.736	6.689	7	6
ZM	ZI311N_1-HE	SRR326797	6.104	6.159	6.159	6.145	6.265	6.167	6	5
ZM	ZI317_1-HE	SRR204011	4.867	4.858	4.913	4.900	4.927	4.893	5	4
ZM	ZI319_2-HE	SRR203461	4.611	4.573	4.622	4.625	4.687	4.624	5	4
ZM	ZI320_1-HE	SRR326793	3.931	3.958	3.902	4.064		3.964	4	3
ZM	ZI324_1-HE	SRR204014	5.986	5.969		6.072	6.074	6.025	6	5
ZM	ZI335_1-HE	SRR203471	5.990	5.959	6.049	6.081	6.062	6.028	6	5
ZM	ZI342_1-HE	SRR094875	6.204	6.148	6.243	6.265	6.395	6.251	6	5
ZM	ZI348_1-HE	SRR203475		5.341	5.359	5.461	5.429	5.398	5	4

Table S8. CNVnator results for strains from six different populations

		Illumina	Reference Genome †						Rounded-Off	CN
**Population ***	**Strain**	**Library ID**	**A4_1**	**A4_2**	**A4_3**	**A4_4**	**A4_5**	**Average**	**Average**	**Estimate ‡**
ZM	ZI353_1-HE	SRR342394	4.387	4.399	4.391	4.411	4.513	4.420	4	3
ZM	ZI358_1-HE	SRR346928	5.868	5.862	5.911	5.930	6.013	5.917	6	5
ZM	ZI362_1-HE_2.2	SRR654685	13.087	12.941	13.123	13.163	13.345	13.132	13	12
ZM	ZI362_1-HE_3.4	SRR346932	12.482	12.762	12.702	12.847	13.192	12.797	13	12
ZI	ZI364_1-HE	SRR204013	5.066		5.111	5.081	5.052	5.078	5	4
ZI	ZI368_1-HE	SRR203462	5.280	5.274	5.289	5.226	5.349	5.284	5	4
ZI	ZI373_1-HE	SRR210782	4.486	4.506	4.595	4.588	4.615	4.558	5	4
ZI	ZI374_1-HE	SRR204008	6.133	6.169	6.234	6.201	6.182	6.184	6	5
ZI	ZI378_1-HE	SRR203464	5.358	5.357		5.384	5.390	5.372	5	4
ZI	ZI381_1-HE	SRR204010		3.665	3.709	3.710	3.736	3.705	4	3
ZI	ZI384_1-HE	SRR354004	4.321	4.332	4.360	4.367	4.421	4.360	4	3
ZI	ZI395_1-HE	SRR326802	4.929	4.936	4.949	4.998	5.084	4.979	5	4
ZI	ZI396_1-HE	SRR353757	4.815	4.867		4.910	4.970	4.890	5	4
ZI	ZI397N_2-HE	SRR654677	7.689	7.690	7.700	7.731	7.841	7.730	8	7
ZI	ZI398_1-HE	SRR346930	4.365	4.362	4.369		4.523	4.405	4	3
ZI	ZI405_2-HE	SRR354003	4.229	4.232	4.233	4.260	4.287	4.248	4	3
ZI	ZI418N_1-HE	SRR202100	5.214	5.235	5.264	5.299	5.305	5.263	5	4
ZI	ZI431_1-HE	SRR654556	4.113	4.105	4.131	4.116	4.254	4.144	4	3
ZI	ZI444_1-HE	SRR203463	4.877	4.846	4.876	4.888	4.957	4.889	5	4
ZI	ZI456_1-HE	SRR203466	5.200	5.189	5.225	5.210	5.263	5.217	5	4
ZI	ZI472_1-HE	SRR203465	7.833	7.890	7.912	7.920	7.968	7.904	8	7
ZI	ZI477_1-HE	SRR353760	4.395	4.385	4.406	4.430	4.527	4.429	4	3
ZI	ZI508_1-HE	SRR346929	3.739		3.794	3.819	3.875	3.807	4	3
ZI	ZI50N_1-HE	SRR203334	6.353	6.372	6.404	6.417	6.517	6.412	6	5
ZI	ZI514N_1-HE	SRR654679		6.137	6.195	6.218	6.301	6.213	6	5
ZI	ZI523_1-HE	SRR342396	3.863	3.868		3.907	3.991	3.907	4	3
ZI	ZI530_1-HE	SRR204009	4.443	4.475	4.517	4.465	4.535	4.487	4	3
ZI	ZI59_1-HE	SRR202112	5.421	5.469	5.469	5.511	5.503	5.475	5	4
ZI	ZI61_1-HE	SRR203472	6.474	6.512	6.537		6.627	6.538	7	6
ZI	ZI85_1-HE	SRR203508	3.989	4.006	4.030	4.031	4.046	4.020	4	3
ZI	ZI99_1-HE	SRR346927		2.917	2.946	3.002	3.010	2.969	3	2

* ZW, Zimbabwe; T, Tasmania; N, The Netherlands; I, Ithaca; B, Beijing (Grenier, et al. 2015). ZM and ZI, Zambia (Lack, et al. 2016b).

† Each reference genome used carries one single *Sdic* copy from the A4 strain and lacks the two parental flanking genes *sw* and *AnxB10*.

The number in the ID of the reference genome denotes the order of the *Sdic* copy in the original strain between the flanking genes, specifically from sw to AnxB10. For example, A4_5 refers to the fifth copy starting from *sw*, *i.e.* the copy adjacent to *AnxB10* in the A4 strain.

Missing values denote not considered values because their associated target size felt outside the expected range, *i.e.* 7.2-8.0 kb.

‡ The CN estimate is calculated as the rounded off average minus one due to the contribution of reads from *sw* and *AnxB10* to the read-depth estimates obtained with CNVnator.

Table S9. Statistical evidence of differences in CN among five populations of the GDL panel

**Contrast**	**Statistic**	***p*-value** *
ZW vs B	D = 8.4508	0.0163
ZW vs I	D = 1.1667	0.9936
ZW vs N	D = 7.2741	0.169
ZW vs T	D = 5.9792	0.2668
I vs B	D = 7.4053	0.042
I vs N	D = -6.1864	0.3212
I vs T	D = -4.0833	0.6452
N vs B	D = 2.5837	0.9282
N vs T	D = 2.3026	0.9593
B vs T	D = 6.1364	0.2116

GDL, Global Diversity Lines (Grenier, et al. 2015).

* According to the Stell-Dwass method.

Table S10. Natural population-specific transposable element (TE) insertions documented in the *Sdic* region

Strain	Size (nt)	Location (from *sw* to *AnxB10*)	Identity *
A2	14,400	Intergenic region downstream *Sdic1*	TE related (*mdg1*)
A5	5,558	*Sdic*_I, intron between exons 2 and 3	TE related (*Tabor*)
A7	17,586	*Sdic*_III, exon 4	TE related (*mdg1, gypsy, jockey*)
B3	5,459	*Sdic*_IV, 3'UTR	TE related (*297*)

* As revealed by BLASTn (Altschul, et al. 1997).

Table S11. Nucleotide differentiation among *Sdic* copies in the reference strain and seven populations from diverse geographic origin of *D. melanogaster*

Whole Repeat (transcriptional Sdic unit + AnxB10-like + defective insertion of the non-LTR retrotransposon Rt1c)

ISO1	ISO1.Sdic2	ISO1.SdicC	ISO1.SdicB	ISO1.Sdic3	ISO1.Sdic4	ISO1.Sdic1	diff.		identity
ISO1.Sdic2		0.00425	0.00522	0.00522	0.00367	0.01301	max	1.38%	98.62%
ISO1.SdicC			0.00367	0.00522	0.00328	0.01380	min	0.27%	99.73%
ISO1.SdicB				0.00270	0.00425	0.01086			
ISO1.Sdic3					0.00541	0.01086			
ISO1.Sdic4						0.01203			
ISO1.Sdic1									

A4	A4.I	A4.II	A4.III	A4.IV	A4.V			diff.	identity
A4.I		0.00514	0.00514	0.00591	0.01418		max	1.42%	98.58%
A4.II			0.00457	0.00457	0.01399		min	0.42%	99.58%
A4.III				0.00419	0.01244				
A4.IV					0.01052				
A4.V									

A5	A5.I.insert	A5.II	A5.III	A5.IV	A5.V			diff.	identity
A5.I.insert		0.00270	0.00541	0.00580	0.01320		max	1.32%	98.68%
A5.II			0.00502	0.00502	0.01320		min	0.19%	99.81%
A5.III				0.00193	0.01203				
A5.IV					0.01281				
A5.V									

A7	A7.I	A7.II	A7.III	A7.IV				diff.	identity
A7.I		0.00480	0.00365	0.01139			max	1.16%	98.84%
A7.II			0.00423	0.01158			min	0.36%	99.64%
A7.III				0.01158					
A7.IV									

B1	B1.I	B1.II	B1.III	B1.IV				diff.	identity
B1.I		0.00540	0.00598	0.01279			max	1.28%	98.72%
B1.II			0.00289	0.01045			min	0.29%	99.71%
B1.III				0.01065					
B1.IV									

B2	B2.I	B2.II	B2.III	B2.IV	B2.V	B2.VI		diff.	identity
B2.I		0.00450	0.00489	0.00587	0.00469	0.01159	max	1.32%	98.68%

Table S11. Nucleotide differentiation among *Sdic* copies in the reference strain and seven populations from diverse geographic origin of *D. melanogaster*

B2.II			0.00234	0.00410	0.00254	0.01258	min	0.23%	99.77%
B2.III				0.00332	0.00332	0.01297			
B2.IV					0.00391	0.01317			
B2.V						0.01317			
B2.VI									

B6	B6.I	B6.II	B6.III		diff.	identity
B6.I		0.00192	0.01082	max	1.08%	98.92%
B6.II			0.01043	min	0.19%	99.81%
B6.III						

B3	B3.I	B3.II	B3.III	B3.IV		diff.	identity
B3.I		0.00444	0.00154	0.02384	max	2.42%	97.58%
B3.II			0.00444	0.02325	min	0.15%	99.85%
B3.III				0.02424			
B3.IV							

Transcriptional Sdic unit only (from promoter to STOP codon)

ISO1	ISO1.Sdic2	ISO1.SdicC	ISO1.SdicB	ISO1.Sdic3	ISO1.Sdic4	ISO1.Sdic1		diff.	identity
ISO1.Sdic2		0.00526	0.00627	0.00551	0.00426	0.01082	max	1.16%	98.84%
ISO1.SdicC			0.00451	0.00577	0.00350	0.01158	min	0.28%	99.72%
ISO1.SdicB				0.00275	0.00501	0.00803			
ISO1.Sdic3					0.00577	0.00778			
ISO1.Sdic4						0.00955			
ISO1.Sdic1									

A4	A4.I	A4.II	A4.III	A4.IV	A4.V		diff.	identity
A4.I		0.00640	0.00640	0.00690	0.01237	max	1.24%	98.76%
A4.II			0.00542	0.00492	0.01187	min	0.49%	99.51%
A4.III				0.00492	0.00988			
A4.IV					0.00789			
A4.V								

A5	A5.I	A5.II	A5.III	A5.IV	A5.V		diff.	identity
A5.I		0.00225	0.00652	0.00677	0.01031	max	1.03%	98.97%
A5.II			0.00526	0.00501	0.00955	min	0.18%	99.83%
A5.III				0.00175	0.00929			

Table S11. Nucleotide differentiation among *Sdic* copies in the reference strain and seven populations from diverse geographic origin of *D. melanogaster*

A5.IV					0.00955				
A5.V									
A7	A7.I	A7.II	A7.III	A7.IV				diff.	identity
A7.I		0.00523	0.00423	0.00924			max	0.95%	99.05%
A7.II			0.00498	0.00898			min	0.42%	99.58%
A7.III				0.00949					
A7.IV									
B1	B1.I	B1.II	B1.III	B1.IV				diff.	identity
B1.I		0.00626	0.00726	0.01054			max	1.05%	98.95%
B1.II			0.00350	0.00776			min	0.35%	99.65%
B1.III				0.00827					
B1.IV									
B2	B2.I	B2.II	B2.III	B2.IV	B2.V	B2.VI		diff.	identity
B2.I		0.00535	0.00611	0.00714	0.00509	0.00868	max	1.07%	98.93%
B2.II			0.00280	0.00484	0.00229	0.00945	min	0.23%	99.77%
B2.III				0.00407	0.00356	0.01022			
B2.IV					0.00407	0.01073			
B2.V						0.00970			
B2.VI									
B6	B6.I	B6.II	B6.III					diff.	identity
B6.I		0.00174	0.00799				max	0.80%	99.20%
B6.II			0.00774				min	0.17%	99.83%
B6.III									
B3	B3.I	B3.II	B3.III	B3.IV				diff.	identity
B3.I		0.00498	0.00149	0.01049			max	1.15%	98.85%
B3.II			0.00498	0.01049			min	0.15%	99.85%
B3.III				0.01150					
B3.IV									

The Jukes-Cantor substitution model was assumed to calculate the level of differentiation between the sequences of each strain in MEGA X (Kumar, et al. 2018). All positions containing gaps and missing data were eliminated (complete deletion option).

The strain ID is highlighted in yellow.

Table S12. RNA-seq datasets examined for expression of *AnxB10*-like

Biological Condition	Run *	Sequencing Reads Considered
Embryos.0.2.hr	SRR1197370	82075821
Embryos.2.4.hr	SRR1197368	32843384
Embryos.4.6.hr	SRR1197338	95071187
Embryos.6.8.hr	SRR1197333	81523580
Embryos.8.10.hr	SRR1197335	82382132
Embryos.10.12.hr	SRR1197367	70050265
Embryos.12.14.hr	SRR1197369	48019376
Embryos.14.16.hr	SRR1197331	77164100
Embryos.16.18.hr	SRR1197330	81995111
Embryos.16.18.hr	SRR1197365	46407303
Embryos.18.20.hr	SRR1197363	46504248
Embryos.20.22.hr	SRR1197364	40376632
Embryos.20.22.hr	SRR1197329	79908102
Embryos.22.24.hr	SRR1197366	40784954
L1.larvae	SRR1197426	64884208
L1.larvae	SRR1197324	89420488
L3.larvae.12.hr.post.molt	SRR1197424	67123887
L3.larvae.PS.1.2	SRR1197312	73465374
L3.larvae.PS.1.2	SRR1197392	67304263
L3.larvae.PS.3.6	SRR1197308	60982886
L3.larvae.PS.3.6	SRR1197388	48598277
L3.larvae.PS.7.9	SRR1197307	72756221
L3.larvae.PS.7.9	SRR1197387	53258332
White.pre.pupae	SRR1197290	77827480
WPP.12.hr	SRR1197289	78985871
WPP.24.hr	SRR1197288	95026533
Pupae.WPP.2.d	SRR1197420	53132443
Pupae.WPP.3.d	SRR1197419	47403639
Pupae.WPP.4.d	SRR1197416	60980117
Adult.female.1.d	SRR1197317	81769224
Adult.male.1.d	SRR1197315	85439694
Adult.female.5.d	SRR1197313	61703967
Adult.female.5.d	SRR1197393	60077629
Adult.male.5.d	SRR1197316	86720278
Adult.female.30.d	SRR1197314	59707987
Adult.female.30.d	SRR1197394	50369484
Adult.male.30.d	SRR1197311	60383756
Adult.male.30.d	SRR1197391	55560405

* (Graveley, et al. 2011).

Table S13. Salient features of the encoded product of Sdic copies annotated in reliable assemblies

Strain_Copy ID	Paratype Group	WD40 Motifs *	Amino Acid Residues	Promoter Class
ISO1_2	*k*	6	543	2
ISO1_C	*l*	6	544	2
ISO1_B	*j*	6	533	4
ISO1_3	*j*	6	533	4
ISO1_4	*c*	4	487	2
ISO1_1	*e*	4	528	2
A4_I	*k*	6	543	1
A4_II	*c*	4	487	2
A4_III	*h*	5	524	1
A4_IV	*c*	4	487	2
A4_V	*e*	4	528	3
A5_I	*j*	6	532	2
A5_II	*b*	4	477	2
A5_III	*g*	5	520	4
A5_IV	*i*	6	539	4
A5_V	*e*	4	527	2
A7_I	*f*	4	456	2
A7_II	*l*	6	544	2
A7_III	*a*	3	388	2
A7_IV	*e*	4	528	2
B1_I	*k*	6	539	1
B1_II	*b*	4	477	2
B1_III	*b*	4	477	1
B1_IV	*e*	4	527	4
B2_I	*k*	6	539	1
B2_II	*b*	4	477	2
B2_III	*b*	4	477	1
B2_IV	*m*	4	434	4
B2_V	*d*	4	495	2
B2_VI	*e*	4	527	2
B3_I	*c*	4	487	2
B3_II	*j*	6	533	2
B3_III	*c*	4	487	2
B3_IV	*e*	4	528	2
B6_V	*l*	6	544	2
B6_II	*f*	4	456	1

Table S13. Salient features of the encoded product of Sdic copies annotated in reliable assemblies

Strain_Copy ID	Paratype Group	WD40 Motifs *	Amino Acid Residues	Promoter Class
B6_III	*e*	4	528	4

* As in WDSPdb, a database for WD40-repeat proteins (Ma, et al. 2019).

Table S14. Gene conversion events detected in the *Sdic* region for eight strains of *D. melanogaster* according to GenConv

		Sim	BC	Coordinates		Offsets	Num	Num	Tot	MisM
Strain	Genes Involved	*p*-value	*p*-value	Begin	End	Len	Poly	Dif	Difs	Pen.
ISO1	ISO1.Berlin.SdicV.4;ISO1.Berlin.SdicVI.1	0	0.00005	1820	3537	1718	49	0	76	None
ISO1	ISO1.Berlin.SdicI.2;ISO1.Berlin.SdicVI.1	0.0006	0.0015	2794	3515	722	37	0	80	None
ISO1	ISO1.Berlin.SdicII.C;ISO1.Berlin.SdicVI.1	0.0001	0.00036	2794	3537	744	38	0	85	None
ISO1	ISO1.Berlin.SdicIII.B;ISO1.Berlin.SdicVI.1	0.0075	0.03696	2874	3537	664	33	0	70	None
ISO1	ISO1.Berlin.SdicIV.3;ISO1.Berlin.SdicVI.1	0.0075	0.03696	2874	3537	664	33	0	70	None
ISO1	ISO1.Berlin.SdicIV.3;ISO1.Berlin.SdicVI.1	0	0	3860	7122	3263	112	0	70	None
ISO1	ISO1.Berlin.SdicIII.B;ISO1.Berlin.SdicVI.1	0.0127	0.05093	3896	4305	410	32	0	70	None
ISO1	ISO1.Berlin.SdicI.2;ISO1.Berlin.SdicVI.1	0.005	0.02089	3992	4305	314	30	0	80	None
ISO1	ISO1.Berlin.SdicII.C;ISO1.Berlin.SdicVI.1	0	0	3992	4498	507	65	0	85	None
ISO1	ISO1.Berlin.SdicII.C;ISO1.Berlin.SdicIV.3	0.0051	0.02221	3992	4498	507	65	0	38	None
ISO1	ISO1.Berlin.SdicI.2;ISO1.Berlin.SdicIII.B	0.0026	0.00849	3992	6437	2446	77	0	35	None
ISO1	ISO1.Berlin.SdicIV.3;ISO1.Berlin.SdicV.4	0	0.00001	3992	7122	3131	108	0	40	None
ISO1	ISO1.Berlin.SdicV.4;ISO1.Berlin.SdicVI.1	0	0	3992	7664	3673	115	0	76	None
ISO1	ISO1.Berlin.SdicI.2;ISO1.Berlin.SdicVI.1	0	0.00005	4314	6437	2124	46	0	80	None
ISO1	ISO1.Berlin.SdicIII.B;ISO1.Berlin.SdicVI.1	0	0	4314	7112	2799	76	0	70	None
ISO1	ISO1.Berlin.SdicIII.B;ISO1.Berlin.SdicV.4	0.0045	0.01846	4314	7112	2799	76	0	33	None
ISO1	ISO1.Berlin.SdicII.C;ISO1.Berlin.SdicVI.1	0	0.00007	4500	7122	2623	42	0	85	None
ISO1	ISO1.Berlin.SdicI.2;ISO1.Berlin.composite	0.0003	0.00087	7280	8675	1396	14	0	169	None
ISO1	ISO1.Berlin.SdicIII.B;ISO1.Berlin.composite	0.0001	0.00047	8335	9096	762	13	0	180	None
ISO1	ISO1.Berlin.SdicIV.3;ISO1.Berlin.composite	0.0005	0.00116	8677	9096	420	12	0	182	None
ISO1	ISO1.Berlin.SdicI.2;ISO1.Berlin.composite	0	0	8729	9559	831	56	0	169	None
ISO1	ISO1.Berlin.SdicII.C;ISO1.Berlin.composite	0.0001	0.00033	9107	9283	177	13	0	182	None
ISO1	ISO1.Berlin.SdicV.4;ISO1.Berlin.composite	0.0009	0.00259	9107	9283	177	13	0	169	None
ISO1	ISO1.Berlin.SdicIV.3;ISO1.Berlin.composite	0	0	9314	9482	169	19	0	182	None
ISO1	ISO1.Berlin.SdicII.C;ISO1.Berlin.composite	0	0	9314	9559	246	29	0	182	None
ISO1	ISO1.Berlin.SdicIII.B;ISO1.Berlin.composite	0	0	9314	9559	246	29	0	180	None
ISO1	ISO1.Berlin.SdicV.4;ISO1.Berlin.composite	0	0	9314	9559	246	29	0	169	None
ISO1	ISO1.Berlin.SdicIV.3;ISO1.Berlin.composite	0.0116	0.0493	9484	9559	76	9	0	182	None
A4	A4.II;A4.V	0.0002	0.00083	2853	3422	570	35	0	86	None

Table S14. Gene conversion events detected in the *Sdic* region for eight strains of *D. melanogaster* according to GenConv

		Sim	BC	Coordinates		Offsets	Num	Num	Tot	MisM
Strain	Genes Involved	*p*-value	*p*-value	Begin	End	Len	Poly	Dif	Difs	Pen.
A4	A4.IV;A4.V	0	0	2853	4390	1538	97	0	65	None
A4	A4.I;A4.V	0.0002	0.00083	2870	3512	643	35	0	86	None
A4	A4.III;A4.V	0.0003	0.00126	2870	3596	727	38	0	78	None
A4	A4.I;A4.V	0.0027	0.00953	3989	4302	314	29	0	86	None
A4	A4.II;A4.V	0	0.00001	3989	4390	402	45	0	86	None
A4	A4.II;A4.IV	0.0061	0.01934	3989	6492	2504	81	0	30	None
A4	A4.III;A4.V	0	0	3989	6493	2505	82	0	78	None
A4	A4.I;A4.IV	0.0085	0.02772	4311	6557	2247	59	0	40	None
A4	A4.I;A4.V	0.0002	0.00083	4392	6492	2101	35	0	86	None
A4	A4.IV;A4.V	0.0114	0.03601	4392	6492	2101	35	0	65	None
A4	A4.II;A4.V	0.0002	0.00055	4392	6493	2102	36	0	86	None
A4	A4.V;A4.composite	0.0133	0.03988	3424	3596	173	6	0	218	None
A4	A4.V;A4.composite	0.0001	0.00014	7278	7777	500	9	0	218	None
A4	A4.III;A4.composite	0.0198	0.05244	8636	8974	339	9	0	180	None
A4	A4.I;A4.composite	0	0	8997	9480	484	39	0	169	None
A4	A4.IV;A4.composite	0.0002	0.00058	9105	9228	124	13	0	178	None
A4	A4.III;A4.composite	0	0.00013	9105	9281	177	14	0	180	None
A4	A4.II;A4.composite	0	0	9312	9480	169	18	0	180	None
A4	A4.III;A4.composite	0	0	9312	9480	169	18	0	180	None
A4	A4.IV;A4.composite	0	0	9312	9480	169	18	0	178	None
A4	A4.II;A4.composite	0.0198	0.05244	9482	9557	76	9	0	180	None
A4	A4.III;A4.composite	0.0198	0.05244	9482	9557	76	9	0	180	None
A4	A4.IV;A4.composite	0.0252	0.06528	9482	9557	76	9	0	178	None
A5	A5.II;A5.V	0.0003	0.00227	2516	3419	904	35	0	80	None
A5	A5.I.insert;A5.V	0.0009	0.00397	2790	3419	630	34	0	79	None
A5	A5.IV;A5.V	0.0051	0.02533	2870	3419	550	29	0	79	None
A5	A5.III;A5.V	0.0112	0.03976	2870	3419	550	29	0	76	None
A5	A5.I.insert;A5.II	0.0014	0.00734	3579	7832	4254	130	0	20	None
A5	A5.II;A5.V	0	0	3986	7271	3286	113	0	80	None

Table S14. Gene conversion events detected in the *Sdic* region for eight strains of *D. melanogaster* according to GenConv

		Sim	BC	Coordinates		Offsets	Num	Num	Tot	MisM
Strain	Genes Involved	*p*-value	*p*-value	Begin	End	Len	Poly	Dif	Difs	Pen.
A5	A5.I.insert;A5.V	0	0	3986	7271	3286	113	0	79	None
A5	A5.III;A5.V	0	0.00002	3988	4389	402	50	0	76	None
A5	A5.IV;A5.V	0.0026	0.01207	4267	4389	123	31	0	79	None
A5	A5.IV;A5.V	0.0002	0.00131	6275	7236	962	37	0	79	None
A5	A5.III;A5.V	0.0003	0.00235	6275	7236	962	37	0	76	None
A5	A5.V;A5.composite	0.045	0.13905	3421	3604	184	5	0	221	None
A5	A5.V;A5.composite	0	0	7274	7874	601	12	0	221	None
A5	A5.III;A5.composite	0.0025	0.0115	7775	8327	553	10	0	181	None
A5	A5.I.insert;A5.composite	0.0001	0.00083	8329	9090	762	12	0	182	None
A5	A5.III;A5.composite	0	0	9308	9476	169	18	0	181	None
A5	A5.I.insert;A5.composite	0	0	9308	9553	246	28	0	182	None
A5	A5.IV;A5.composite	0	0	9308	9553	246	28	0	178	None
A5	A5.II;A5.composite	0	0	9308	9553	246	28	0	174	None
A5	A5.III;A5.composite	0.0109	0.03957	9478	9553	76	9	0	181	None
A7	A7.II;A7.IV	0.0004	0.00194	2304	3398	1095	37	0	73	None
A7	A7.III;A7.IV	0	0.00013	2304	3571	1268	44	0	74	None
A7	A7.I;A7.IV	0.0001	0.00067	2315	3515	1201	42	0	70	None
A7	A7.II;A7.IV	0	0.00001	3989	4384	396	54	0	73	None
A7	A7.I;A7.IV	0	0.00002	3991	4384	394	53	0	70	None
A7	A7.III;A7.IV	0	0	3991	4438	448	65	0	74	None
A7	A7.I;A7.II	0	0.00009	3991	7209	3219	113	0	31	None
A7	A7.III;A7.IV	0.0006	0.00322	6256	7209	954	35	0	74	None
A7	A7.II;A7.IV	0.0006	0.00388	6256	7209	954	35	0	73	None
A7	A7.I;A7.IV	0.0006	0.00484	6256	7228	973	36	0	70	None
A7	A7.I;A7.composite	0.0222	0.06146	3428	4128	701	9	0	169	None
A7	A7.II;A7.composite	0.0297	0.07748	7266	7844	579	8	0	178	None
A7	A7.IV;A7.composite	0	0	7266	8661	1396	14	0	220	None
A7	A7.III;A7.composite	0	0.00026	8663	9082	420	12	0	182	None
A7	A7.I;A7.composite	0	0	8715	9269	555	26	0	169	None

Table S14. Gene conversion events detected in the *Sdic* region for eight strains of *D. melanogaster* according to GenConv

		Sim	BC	Coordinates		Offsets	Num	Num	Tot	MisM
Strain	Genes Involved	*p*-value	*p*-value	Begin	End	Len	Poly	Dif	Difs	Pen.
A7	A7.III;A7.composite	0	0.00002	9084	9269	186	14	0	182	None
A7	A7.III;A7.composite	0	0	9300	9468	169	18	0	182	None
A7	A7.I;A7.composite	0	0	9300	9468	169	18	0	169	None
A7	A7.II;A7.composite	0	0	9300	9545	246	28	0	178	None
A7	A7.III;A7.composite	0.0036	0.01345	9470	9545	76	9	0	182	None
A7	A7.I;A7.composite	0.0222	0.06146	9470	9545	76	9	0	169	None
B1	B1.I;B1.IV	0.0007	0.00391	2853	3138	286	32	0	78	None
B1	B1.III;B1.IV	0.0018	0.00631	2853	3632	780	37	0	66	None
B1	B1.II;B1.IV	0.0054	0.01609	2870	3666	797	34	0	66	None
B1	B1.II;B1.IV	0	0	3668	7740	4073	121	0	66	None
B1	B1.I;B1.IV	0.002	0.00838	3985	4300	316	30	0	78	None
B1	B1.III;B1.IV	0.0002	0.00038	3985	4388	404	46	0	66	None
B1	B1.I;B1.IV	0.0408	0.12025	4390	6270	1881	23	0	78	None
B1	B1.III;B1.IV	0	0	4390	7268	2879	64	0	66	None
B1	B1.II;B1.composite	0.0007	0.00314	7271	8319	1049	11	0	172	None
B1	B1.IV;B1.composite	0.0002	0.00032	8187	8919	733	7	0	220	None
B1	B1.I;B1.composite	0	0	8715	9545	831	56	0	170	None
B1	B1.II;B1.composite	0.037	0.11466	9147	9268	122	8	0	172	None
B1	B1.III;B1.composite	0	0	9299	9545	247	28	0	179	None
B1	B1.II;B1.composite	0	0	9299	9545	247	28	0	172	None
B2	B2.V;B2.VI	0.0004	0.00248	2658	3419	762	36	0	79	None
B2	B2.II;B2.VI	0.0008	0.00523	2790	3419	630	35	0	77	None
B2	B2.I;B2.VI	0.0297	0.1333	2870	3138	269	28	0	72	None
B2	B2.IV;B2.VI	0.0036	0.01686	2870	3419	550	30	0	81	None
B2	B2.III;B2.VI	0.0049	0.02321	2870	3419	550	30	0	79	None
B2	B2.III;B2.V	0.0042	0.01981	3723	7236	3514	116	0	21	None
B2	B2.I;B2.VI	0.0096	0.04914	3986	4301	316	31	0	72	None
B2	B2.II;B2.VI	0	0.00007	3986	4389	404	47	0	77	None
B2	B2.III;B2.VI	0	0	3986	6273	2288	70	0	79	None

Table S14. Gene conversion events detected in the *Sdic* region for eight strains of *D. melanogaster* according to GenConv

		Sim	BC	Coordinates		Offsets	Num	Num	Tot	MisM
Strain	Genes Involved	*p*-value	*p*-value	Begin	End	Len	Poly	Dif	Difs	Pen.
B2	B2.V;B2.VI	0	0	3986	6273	2288	70	0	79	None
B2	B2.IV;B2.VI	0	0	3988	6273	2286	69	0	81	None
B2	B2.III;B2.IV	0.0037	0.01705	3988	7236	3249	108	0	23	None
B2	B2.IV;B2.V	0.0007	0.00432	3988	7658	3671	113	0	25	None
B2	B2.I;B2.VI	0.0007	0.00477	4310	6273	1964	38	0	72	None
B2	B2.I;B2.IV	0.001	0.00581	4310	7116	2807	73	0	38	None
B2	B2.I;B2.III	0.0057	0.02681	4310	7116	2807	73	0	33	None
B2	B2.I;B2.V	0.0057	0.02681	4310	7116	2807	73	0	33	None
B2	B2.II;B2.VI	0	0	4391	7271	2881	62	0	77	None
B2	B2.I;B2.VI	0.0041	0.01809	6275	7116	842	34	0	72	None
B2	B2.IV;B2.VI	0	0.00078	6275	7236	962	38	0	81	None
B2	B2.V;B2.VI	0	0.00118	6275	7236	962	38	0	79	None
B2	B2.III;B2.VI	0	0.00081	6275	7271	997	39	0	79	None
B2	B2.VI;B2.composite	0.0056	0.02656	3606	3802	197	6	0	220	None
B2	B2.II;B2.composite	0.001	0.00532	7274	8322	1049	12	0	171	None
B2	B2.I;B2.composite	0	0	8666	9548	883	56	0	165	None
B2	B2.IV;B2.composite	0	0	9150	9471	322	27	0	181	None
B2	B2.III;B2.composite	0	0	9302	9548	247	29	0	172	None
B2	B2.II;B2.composite	0	0	9302	9548	247	29	0	171	None
B2	B2.V;B2.composite	0	0	9303	9548	246	28	0	169	None
B2	B2.IV;B2.composite	0.0113	0.05079	9473	9548	76	9	0	181	None
B3	B3.II;B3.IV.insertion	0	0.00004	2300	2988	689	29	0	142	None
B3	B3.III;B3.IV.insertion	0	0.00013	2443	2988	546	26	0	147	None
B3	B3.I;B3.IV.insertion	0.0009	0.00174	2790	2988	199	22	0	146	None
B3	B3.II;B3.IV.insertion	0	0	4016	4411	396	55	0	142	None
B3	B3.III;B3.IV.insertion	0	0	4018	4411	394	54	0	147	None
B3	B3.I;B3.IV.insertion	0	0	4018	6641	2624	106	0	146	None
B3	B3.II;B3.III	0.0003	0.0012	4018	7624	3607	117	0	32	None
B3	B3.III;B3.IV.insertion	0	0	4413	6499	2087	34	0	147	None

Table S14. Gene conversion events detected in the *Sdic* region for eight strains of *D. melanogaster* according to GenConv

		Sim	BC	Coordinates		Offsets	Num	Num	Tot	MisM
**Strain**	**Genes Involved**	***p*-value**	***p*-value**	**Begin**	**End**	**Len**	**Poly**	**Dif**	**Difs**	**Pen.**
B3	B3.II;B3.IV.insertion	0	0	4413	6499	2087	34	0	142	None
B3	B3.III;B3.IV.insertion	0.0197	0.06563	6501	6641	141	16	0	147	None
B3	B3.II;B3.IV.insertion	0.0319	0.1009	6501	6641	141	16	0	142	None
B3	B3.I;B3.II	0.0045	0.01443	9333	9600	268	91	0	33	None
B3	B3.II;B3.III	0.0064	0.01928	9333	9600	268	91	0	32	None
B3	B3.IV.insertion;B3.composite	0.003	0.00901	3291	3634	344	5	0	291	None
B3	B3.IV.insertion;B3.composite	0.0414	0.11335	7298	7749	452	4	0	291	None
B3	B3.III;B3.composite	0.007	0.01974	9126	9249	124	14	0	176	None
B3	B3.I;B3.composite	0	0	9333	9504	172	65	0	179	None
B3	B3.II;B3.composite	0	0	9333	9504	172	65	0	179	None
B3	B3.III;B3.composite	0	0	9333	9504	172	65	0	176	None
B3	B3.I;B3.composite	0	0	9506	9600	95	25	0	179	None
B3	B3.II;B3.composite	0	0	9506	9600	95	25	0	179	None
B3	B3.III;B3.composite	0	0	9506	9600	95	25	0	176	None
B6	B6.II;B6.III	0.0002	0.00109	2857	3581	725	38	0	67	None
B6	B6.I;B6.III	0.0014	0.00577	2874	3426	553	32	0	69	None
B6	B6.I;B6.III	0	0.00002	3896	4385	490	48	0	69	None
B6	B6.II;B6.III	0.0018	0.01154	4286	4385	100	31	0	67	None
B6	B6.I;B6.II	0.0087	0.03597	4286	8873	4588	100	0	18	None
B6	B6.I;B6.III	0	0	4387	8576	4190	66	0	69	None
B6	B6.II;B6.III	0	0	4387	8576	4190	66	0	67	None
B6	B6.III;B6.composite	0.0004	0.00142	8250	8916	667	5	0	219	None
B6	B6.II;B6.composite	0	0.00019	8660	9079	420	11	0	177	None
B6	B6.II;B6.composite	0.0185	0.0538	9144	9213	70	7	0	177	None
B6	B6.I;B6.composite	0.0033	0.01503	9144	9266	123	8	0	176	None
B6	B6.II;B6.composite	0	0	9297	9465	169	18	0	177	None
B6	B6.I;B6.composite	0	0	9297	9465	169	18	0	176	None
B6	B6.II;B6.composite	0.0009	0.00321	9467	9542	76	9	0	177	None

Table S14. Gene conversion events detected in the *Sdic* region for eight strains of *D. melanogaster* according to GenConv

		Sim	BC	Coordinates		Offsets	Num	Num	Tot	MisM
**Strain**	**Genes Involved**	***p*-value**	***p*-value**	**Begin**	**End**	**Len**	**Poly**	**Dif**	**Difs**	**Pen.**
B6	B6.I;B6.composite	0.0011	0.00373	9467	9542	76	9	0	176	None

Only inner fragments (or events) are considered.

Sim *p*-value, probability based on 10,000 permutations.

BC *p*-value, Bonferroni-corrected KA (BLAST-like) *p*-values.

Len, tract length.

Num Poly, number of polymorphic sites in the fragment.

Num Dif, number of mismatches within the fragment.

Tot Difs, total number of mismatches between two sequences.

MisM Pen, penalty per mismatch for the two sequences involved.

Table S15. Evolution mode across partitions of the *Sdic* repeat as delineated with ACG

Partition *	Branch †	LRT	*P*-value (FDR)	Node and Subtree †
P1	36	14.647	0.000129654	[Node6] Internal Branch Rooting (A7_IV,(A4_IV,(ISO1_Berlin_SdicV_4,(((((ISO1_Berlin_SdicVI_1,A5_V)Node16,A4_V)Node15,B3_IV_insertion)Node14,(B6_III,((B2_IV,B1_IV)Node24,(((B6_II,(A5_II,(ISO1_Berlin_SdicIV_3,(B2_III,B1_III)Node35)Node33)Node31)Node29,(B6_I,(A7_II,A7_I)Node40)Node38)Node28,A5_IV)Node27)Node23)Node21)Node13,((A4_I,(A5_I_insert,A5_III)Node47)Node45,((B2_V,((((((B3_I,(B3_III,(A7_III,B3_II)Node62)Node60)Node58,(ISO1_Berlin_SdicII_C,ISO1_Berlin_SdicIII_B)Node65)Node57,ISO1_Berlin_SdicI_2)Node56,A4_II)Node55,((A4_III,B1_I)Node71,B2_I)Node70)Node54,(B2_II,B1_II)Node75)Node53)Node51,B2_VI)Node50)Node44)Node12)Node10)Node8)Node6
P1.2	36	16.467	4.95E-05	[Node3] Internal Branch Rooting ((B3_II,(((B2_IV,(B6_III,B1_IV)Node10)Node8,(B3_III,A7_III)Node13)Node7,((((B2_III,((A7_II,(A5_IV,ISO1_Berlin_SdicIV_3)Node24)Node22,B6_I)Node21)Node19,(B6_II,B1_III)Node28)Node18,A7_I)Node17,A5_II)Node16)Node6)Node4,((((((ISO1_Berlin_SdicVI_1,A4_V)Node38,ISO1_Berlin_SdicV_4)Node37,A5_I_insert)Node36,B1_II)Node35,((A5_III,B2_VI)Node45,(((B2_V,(((A4_II,(A4_IV,ISO1_Berlin_SdicI_2)Node56)Node54,(ISO1_Berlin_SdicII_C,A7_IV)Node59)Node53,B2_I)Node52)Node50,(A4_I,A5_V)Node63)Node49,((ISO1_Berlin_SdicIII_B,((B1_I,B3_IV_insertion)Node70,B3_I)Node69)Node67,B2_II)Node66)Node48)Node44)Node34,A4_III)Node33)Node3
P2	39	229.270	< 1.00E-010	[Node75] Internal Branch Rooting (A4_composite,(ISO1_Berlin_composite,((A7_composite,A5_composite)Node80,(B6_composite,(B2_composite,B1_composite)Node85)Node83)Node79)Node77)Node75
P2	53	228.391	< 1.00E-010	[B1_III] Leaf node B1_III
P2	58	18.306	1.88E-05	[ISO1_Berlin_SdicVI_1] Leaf node ISO1_Berlin_SdicVI_1
P2	62	19.362	0.000010816	[ISO1_Berlin_SdicI_2] Leaf node ISO1_Berlin_SdicI_2
P2	64	228.391	< 1.00E-010	[A5_I_insert] Leaf node A5_I_insert
P2	67	227.341	< 1.00E-010	[B2_II] Leaf node B2_II
P2	83	228.390	< 1.00E-010	[A7_composite] Leaf node A7_composite
P3	43	47.982	< 1.00E-010	[Node16] Internal Branch Rooting ((((B1_IV,A5_III)Node19,(ISO1_Berlin_SdicIV_3,(ISO1_Berlin_SdicIII_B,(B6_III,(A5_IV,B2_IV)Node28)Node26)Node24)Node22)Node18,((A4_III,B6_II)Node32,(((A4_I,B1_III)Node37,(B2_I,B1_I)Node40)Node36,B2_III)Node35)Node31)Node17,((ISO1_Berlin_SdicV_4,(B3_III,(A7_III,(((((B2_VI,A4_IV)Node55,B2_II)Node54,(ISO1_Berlin_SdicII_C,A4_II)Node59)Node53,(A7_II,A7_IV)Node62)Node52,(((((((ISO1_Berlin_SdicI_2,B6_I)Node71,(ISO1_Berlin_SdicVI_1,B1_II)Node74)Node70,B3_I)Node69,A5_II)Node68,B3_II)Node67,(A7_I,(B3_IV_insertion,(A5_I_insert,A5_V)Node84)Node82)Node80)Node66,B2_V)Node65)Node51)Node49)Node47)Node45,A4_V)Node44)Node16
P3	7	33.020	9.10E-09	[Node1] Internal Branch Rooting ((((ISO1_Berlin_composite,A4_composite)Node4,B6_composite)Node3,A7_composite)Node2,(A5_composite,((B2_composite,B1_composite)Node12,B3_composite)Node11)Node9)Node1
P3	89	17.598	2.73E-05	[simulans_composite] Leaf node simulans_composite
P5	43	26.670	2.41E-07	Node16] Internal Branch Rooting ((B3_III,(((A5_IV,(A5_III,B2_V)Node23)Node21,A7_IV)Node20,(A7_II,(ISO1_Berlin_SdicII_C,((B6_II,B6_I)Node32,((A4_II,((B3_I,A4_IV)Node39,((((ISO1_Berlin_SdicVI_1,(((B2_IV,(ISO1_Berlin_SdicIV_3,((ISO1_Berlin_SdicIII_B,(A5_I_insert,A4_III)Node56)Node54,(A7_III,B3_II)Node59)Node53)Node51)Node49,B2_III)Node48,(B1_III,A5_II)Node63)Node47)Node45,(ISO1_Berlin_SdicV_4,(A4_V,(B3_IV_insertion,A5_V)Node70)Node68)Node66)Node44,(B2_VI,B1_IV)Node73)Node43,(A7_I,(B2_I,(A4_I,(ISO1_Berlin_SdicI_2,B1_I)Node82)Node80)Node78)Node76)Node42)Node38)Node36,B6_III)Node35)Node31)Node29)Node27)Node19)Node17,(B2_II,B1_II)Node86)Node16
P5	7	15.954	6.49E-05	[Node1] Internal Branch Rooting (A4_composite,((B1_composite,(((A7_composite,B3_composite)Node8,B2_composite)Node7,(B6_composite,A5_c

Table S15. Evolution mode across partitions of the *Sdic* repeat as delineated with ACG

Partition *	Branch †	LRT	*P*-value (FDR)	Node and Subtree †
				omposite)Node12)Node6)Node4,ISO1_Berlin_composite)Node3)Node1
P5.0a	43	65.480	< 1.00E-010	[Node16] Internal Branch Rooting (A4_II,((((A4_I,(ISO1_Berlin_SdicI_2,B1_I)Node23)Node21,B2_I)Node20,(ISO1_Berlin_SdicIV_3,ISO1_Berlin_SdicII_C)Node27)Node19,((((A7_III,(((B3_IV_insertion,B3_I)Node37,(((A5_I_insert,B1_IV)Node42,(A5_V,A5_II)Node45)Node41,(B2_II,B1_II)Node48)Node40)Node36,(((A7_IV,A4_V)Node53,B6_III)Node52,A4_III)Node51)Node35)Node33,(B2_VI,B1_III)Node58)Node32,(B2_III,((((ISO1_Berlin_SdicVI_1,((B3_II,(A7_II,ISO1_Berlin_SdicV_4)Node71)Node69,B3_III)Node68)Node66,(((A5_IV,A7_I)Node77,B6_I)Node76,(A5_III,B6_II)Node81)Node75)Node65,B2_V)Node64,B2_IV)Node63)Node61)Node31,(ISO1_Berlin_SdicIII_B,A4_IV)Node86)Node30)Node18)Node16
P5.0a	7	25.591	4.22E-07	[Node1] Internal Branch Rooting ((((B2_composite,((B3_composite,A7_composite)Node7,(B6_composite,B1_composite)Node10)Node6)Node4,A5_composite)Node3,ISO1_Berlin_composite)Node2,A4_composite)Node1
P6	42	22.381	2.24E-06	[Node76] Internal Branch Rooting ((((B1_IV,B6_III)Node79,B2_VI)Node78,(A4_V,(A7_IV,ISO1_Berlin_SdicVI_1)Node85)Node83)Node77,A5_V)Node76
P6.2	42	32.350	1.29E-08	[Node75] Internal Branch Rooting (ISO1_Berlin_SdicVI_1,((((B2_VI,A4_V)Node80,A7_IV)Node79,A5_V)Node78,(B6_III,B1_IV)Node85)Node77)Node75
P6.2	88	236.574	< 1.00E-010	[B3_IV_insertion] Leaf node B3_IV_insertion

***** As shown in Fig. S8.
† The branch number in Hyphy and its associated node and subtree are relative to the PARTITION SPECIFIC gene tree.

Table S16. Statistical support for differences in *Sdic male* expression according to qRT-PCR experiments

Contrast	Statistic	*P*
Set 1	F= 61.7304	<0.0001 *
ISO-1 vs 4M	D = 1.0075	<0.0002 †
ISO-1 vs 2T	D = 0.5150	0.0278 †
ISO-1 vs w^{1118}	D = 1.0150	0.0001 †
w^{1118} vs 4M	D = 2.0225	<0.0001 †
w^{1118} vs 2T	D = 1.5300	<0.0001 †
2T vs 4M	D = 0.4925	0.0358 †
Set 2	F = 9.9913	<0.0001 *
ISO-1 vs B3	D = 0.2875	0.7309 †
ISO-1 vs OR-R	D = 0.3800	0.4387 †
ISO-1 vs A4	D = 0.5575	0.0924 †
ISO-1 vs B6	D = 0.8725	0.0025 †
ISO-1 vs B2	D = 0.9125	0.0015 †
ISO-1 vs A7	D = 1.2275	<0.0001 †
OR-R vs A4	D = 0.1775	0.9617 †
OR-R vs B6	D = 0.4925	0.1751 †
OR-R vs B2	D = 0.5235	0.1190 †
OR-R vs A7	D = 0.8475	0.0033 †
B3 vs OR-R	D = 0.0925	0.9987 †
B3 vs A4	D = 0.2700	0.7818 †
B3 vs B6	D = 0.5850	0.0693 †
B3 vs B2	D = 0.6250	0.0449 †
B3 vs A7	D = 0.9400	0.0011 †
A4 vs B6	D = 0.3150	0.6449 †
A4 vs B2	D = 0.3550	0.5161 †
A4 vs A7	D = 0.6700	0.0271 †
B6 vs B2	D = 0.0400	1.0000 †
B6 vs A7	D = 0.3550	0.5161 †
B2 vs A7	D = 0.3150	0.6449 †

* One-way ANOVA test.
† Tukey-Kramer HSD post-hoc test.

Table S17. Statistical support for differences in sperm competitive ability in offense assays

**Competing Experimental Males**	**Statistic**	***P*** *
Strain set 1		
w^{1118} vs 2T	D = 5.0432	0.6627
w^{1118} vs A^-	D = 18.4266	0.0007
w^{1118} vs B^+	D = 5.1064	0.625
B^+ vs 2T	D = -0.6481	0.9991
B^+ vs A^-	D = 13.9056	0.0263
A^- vs 2T	D = -15.8999	0.0103
Strain set 2		
w^{1118} vs 4M	D = 19.1928	0.0016
w^{1118} vs E^-	D =18.8472	0.0062
w^{1118} vs I^+	D = 1.4688	0.9897
I^+ vs 4M	D = 17.7019	0.0048
I^+ vs E^-	D = 16.7507	0.0166
E^- vs 4M	D = 6.3411	0.7216

* According to the Stell-Dwass method.

References Cited

Abyzov A, Urban AE, Snyder M, Gerstein M 2011. CNVnator: An approach to discover, genotype, and characterize typical and atypical CNVs from family and population genome sequencing. Genome research 21: 974-984.

Adams MD, Celniker SE, Holt RA, Evans CA, Gocayne JD, Amanatides PG, Scherer SE, Li PW, Hoskins RA, Galle RF, *et al.* 2000. The genome sequence of *Drosophila melanogaster*. Science 287: 2185-2195.

Altschul SF, Madden TL, Schaffer AA, Zhang J, Zhang Z, Miller W, Lipman DJ 1997. Gapped BLAST and PSI-BLAST: a new generation of protein database search programs. Nucleic Acids Res 25: 3389-3402.

Benjamini Y, Hochberg Y 1995. Controlling the False Discovery Rate - a Practical and Powerful Approach to Multiple Testing. Journal of the Royal Statistical Society Series B-Methodological 57: 289-300.

Berlin K, Koren S, Chin CS, Drake JP, Landolin JM, Phillippy AM 2015. Assembling large genomes with single-molecule sequencing and locality-sensitive hashing. Nature biotechnology 33: 623-630.

Bray JR, Curtis JT 1957. An Ordination of the Upland Forest Communities of Southern Wisconsin. Ecological Monographs 27: 326-349.

Chakraborty M, Baldwin-Brown JG, Long AD, Emerson JJ 2016. Contiguous and accurate de novo assembly of metazoan genomes with modest long read coverage. Nucleic Acids Res.

Chakraborty M, Emerson JJ, Macdonald SJ, Long AD 2019. Structural variants exhibit widespread allelic heterogeneity and shape variation in complex traits. Nat Commun 10: 4872.

Chakraborty M, VanKuren NW, Zhao R, Zhang X, Kalsow S, Emerson JJ 2018. Hidden genetic variation shapes the structure of functional elements in Drosophila. Nat Genet 50: 20-25.

Clifton BD, Librado P, Yeh SD, Solares ES, Real DA, Jayasekera SU, Zhang W, Shi M, Park RV, Magie RD, *et al.* 2017. Rapid Functional and Sequence Differentiation of a Tandemly Repeated Species-Specific Multigene Family in Drosophila. Mol Biol Evol 34: 51-65.

dos Santos G, Schroeder AJ, Goodman JL, Strelets VB, Crosby MA, Thurmond J, Emmert DB, Gelbart WM, FlyBase C 2015. FlyBase: introduction of the Drosophila melanogaster Release 6 reference genome assembly and large-scale migration of genome annotations. Nucleic Acids Res 43: D690-697.

Graveley BR, Brooks AN, Carlson JW, Duff MO, Landolin JM, Yang L, Artieri CG, van Baren MJ, Boley N, Booth BW, *et al.* 2011. The developmental transcriptome of Drosophila melanogaster. Nature 471: 473-479.

Grenier JK, Arguello JR, Moreira MC, Gottipati S, Mohammed J, Hackett SR, Boughton R, Greenberg AJ, Clark AG 2015. Global diversity lines - a five-continent reference panel of sequenced Drosophila melanogaster strains. G3 (Bethesda, Md) 5: 593-603.

Hu TT, Eisen MB, Thornton KR, Andolfatto P 2013. A second-generation assembly of the Drosophila simulans genome provides new insights into patterns of lineage-specific divergence. Genome research 23: 89-98.

Kim KE, Peluso P, Babayan P, Yeadon PJ, Yu C, Fisher WW, Chin CS, Rapicavoli NA, Rank DR, Li J, *et al.* 2014. Long-read, whole-genome shotgun sequence data for five model organisms. Sci Data 1: 140045.

King EG, Macdonald SJ, Long AD 2012a. Properties and power of the Drosophila Synthetic Population Resource for the routine dissection of complex traits. Genetics 191: 935-949.

King EG, Merkes CM, McNeil CL, Hoofer SR, Sen S, Broman KW, Long AD, Macdonald SJ 2012b. Genetic dissection of a model complex trait using the Drosophila Synthetic Population Resource. Genome research 22: 1558-1566.
Kumar S, Stecher G, Li M, Knyaz C, Tamura K 2018. MEGA X: Molecular Evolutionary Genetics Analysis across Computing Platforms. Mol Biol Evol 35: 1547-1549.
Lack JB, Lange JD, Tang AD, Corbett-Detig RB, Pool JE 2016a. A Thousand Fly Genomes: An Expanded Drosophila Genome Nexus. Mol Biol Evol 33: 3308-3313.
Lack JB, Lange JD, Tang AD, Corbett-Detig RB, Pool JE 2016b. A Thousand Fly Genomes: An Expanded Drosophila Genome Nexus. Mol Biol Evol 33: 3308-3313.
Langley CH, Stevens K, Cardeno C, Lee YC, Schrider DR, Pool JE, Langley SA, Suarez C, Corbett-Detig RB, Kolaczkowski B*, et al.* 2012. Genomic variation in natural populations of Drosophila melanogaster. Genetics 192: 533-598.
Ma J, An K, Zhou JB, Wu NS, Wang Y, Ye ZQ, Wu YD 2019. WDSPdb: an updated resource for WD40 proteins. Bioinformatics (Oxford, England).
Nurminsky DI, Nurminskaya MV, De Aguiar D, Hartl DL 1998. Selective sweep of a newly evolved sperm-specific gene in Drosophila. Nature 396: 572-575.
Parks AL, Cook KR, Belvin M, Dompe NA, Fawcett R, Huppert K, Tan LR, Winter CG, Bogart KP, Deal JE*, et al.* 2004. Systematic generation of high-resolution deletion coverage of the Drosophila melanogaster genome. Nat Genet 36: 288-292.
Ranz J, Clifton B 2019. Characterization and evolutionary dynamics of complex regions in eukaryotic genomes. Sci China Life Sci.
Ryder E, Blows F, Ashburner M, Bautista-Llacer R, Coulson D, Drummond J, Webster J, Gubb D, Gunton N, Johnson G*, et al.* 2004. The DrosDel collection: a set of P-element insertions for generating custom chromosomal aberrations in Drosophila melanogaster. Genetics 167: 797-813.
Sawyer S 1989. Statistical tests for detecting gene conversion. Mol Biol Evol 6: 526-538.
Solares EA, Chakraborty M, Miller DE, Kalsow S, Hall K, Perera AG, Emerson JJ, Hawley RS 2018. Rapid Low-Cost Assembly of the Drosophila melanogaster Reference Genome Using Low-Coverage, Long-Read Sequencing. G3 (Bethesda, Md) 8: 3143-3154.
Yeh SD, Do T, Abbassi M, Ranz JM 2012a. Functional relevance of the newly evolved sperm dynein intermediate chain multigene family in Drosophila melanogaster males. Commun Integr Biol 5: 462-465.
Yeh SD, Do T, Chan C, Cordova A, Carranza F, Yamamoto EA, Abbassi M, Gandasetiawan KA, Librado P, Damia E*, et al.* 2012b. Functional evidence that a recently evolved Drosophila sperm-specific gene boosts sperm competition. Proceedings of the National Academy of Sciences of the United States of America 109: 2043-2048.
